# Spins Afar—Long-Range Pulse Dipolar EPR Spectroscopy Uncovers Curvature in TRIM25’s Coiled Coil

**DOI:** 10.1021/acs.jpclett.6c02671

**Published:** 2026-10-05

**Authors:** Yannik Limbach, Katrin Ackermann, Aisika Chakraborty, David G. Norman, Bela E. Bode

**Affiliations:** † EaStCHEM School of Chemistry, Biomedical Sciences Research Complex and Centre of Magnetic Resonance, 7486 University of St.Andrews, North Haugh, St Andrews KY16 9ST, 248 Scotland UK; ‡ Clausius-Institute for Physical and Theoretical Chemistry, University of Bonn, Wegelerstr. 12, 53115 Bonn, Germany; §School of Life Sciences, 3042 University of Dundee, Medical Sciences Institute, Nethergate Dundee DD1 4HN, Scotland, UK

## Abstract

Pulse Dipolar Electron Paramagnetic Resonance Spectroscopy (PDS) is a powerful method for determining nanometer distances in biomolecular systems. This methodology has been successfully used to validate structure hypotheses and to identify disorder. However, inherent distance limitations are under-explored. Many challenging biomolecular applications require long distance information, driving the need for a benchmarking study. We demonstrate distances up to 10.5 nm in TRIM25 reliably extracted from data obtained under standard sample conditions. By protein perdeuteration accurate distance measurements up to 17.3 nm were enabled. Enhancing sensitivity through optimization of the measurement and sample preparation conditions the achievable dipolar evolution time was extended to 150 μs. Thus, distances close to 20 nm should become experimentally accessible, highlighting the potential of advanced PDS methodologies for probing previously inaccessible long-range structural information in complex systems.

## Graphical abstract

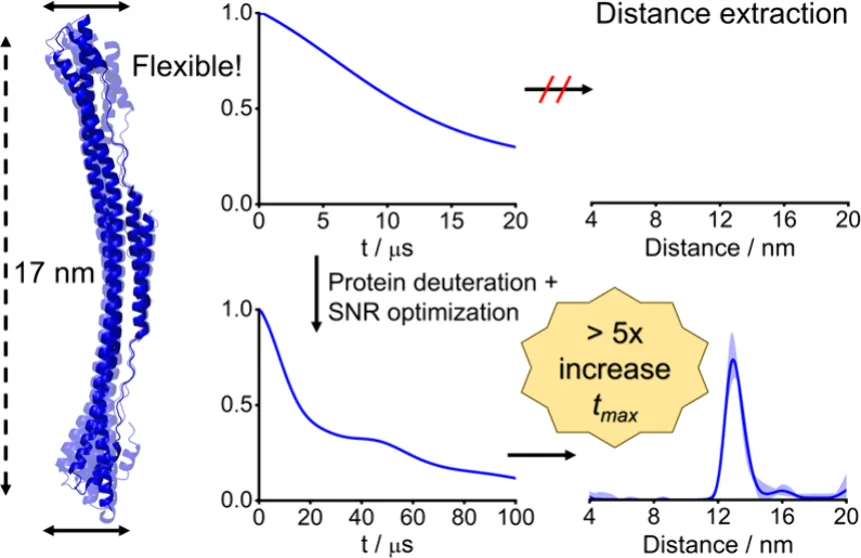

An interesting target for long distance determination is the tripartite motif (TRIM) protein superfamily forming a part of the group of real interesting new gene (RING) E3 ubiquitin ligases.^1^ These are involved in post-translational modifications such as ubiquitination,^2^ regulation of immunity and as viral restriction factors.^3^ Particularly interesting is the coiled coil domain of these proteins (Figure 1a), due to its unusual antiparallel arrangement and length of about 18 nm.^4^ This highly stable dimeric arrangement allows for convenient placing of two spins with increasing distances. Strikingly, the reported structures of this domain show differing degrees of curvature of the coiled coil (e.g., TRIM5, TRIM69 and TRIM25),^5–7^ making them intriguing targets for Pulse Dipolar Electron Paramagnetic Resonance Spectroscopy (PDS) distance measurements to unravel the curvature and conformational distribution in solution.

**Figure 1:**
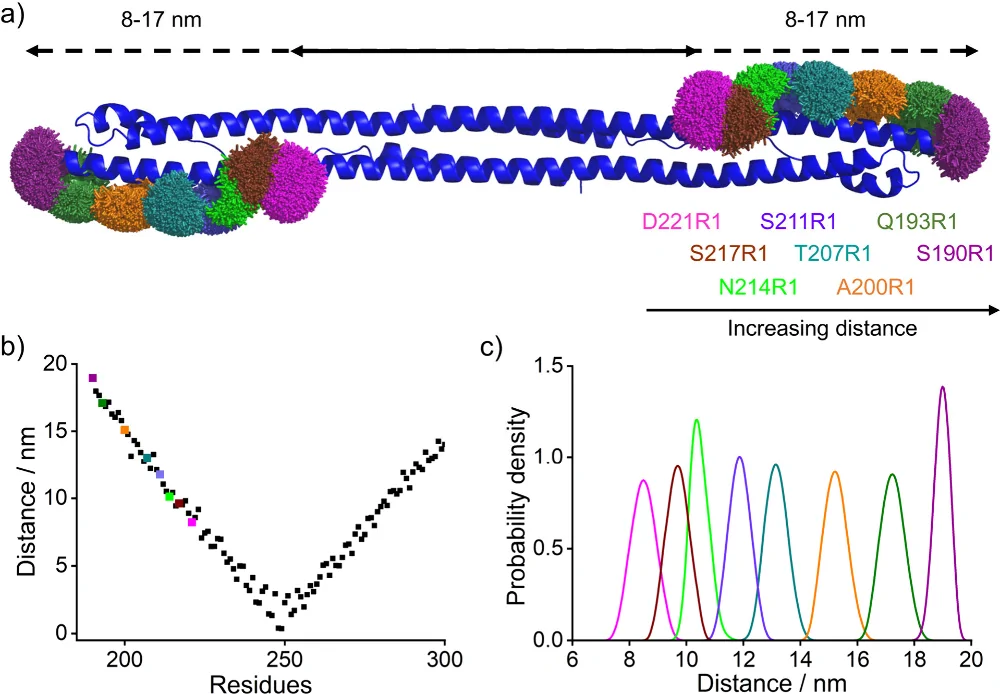
In-silico mutant design based on a crystal structure (PDB: 4ltb^7^) of the dimeric antiparallel coiled coil domain (residues 189–379) of TRIM25 with ChiLife.^46^ (a) Visualization of the crystal structure with the MTSL rotamer clouds and (b) average distances between the residues of the coiled coil region (residues 189–300) calculated in ChiLife. Selected residues for spin label insertion were highlighted in the same color code as in (a). (c) Distance distributions of the selected residues in the same color code as in (a), predicted via the off-rotamer (OR) approach in ChiLife.

The combination of site-directed spin labeling (SDSL)^8^ of proteins and PDS^9,10^ has provided valuable insight into conformational states and equilibria within proteins^11–14^ and nucleic acids,^15–17^ structured and nonstructured domains^18–20^ and arrangement of larger protein complexes.^21–24^ The most broadly applied technique for this is the pulsed electron–electron double resonance (PELDOR/DEER) method.^25–28^ Commonly, systems labeled with commercially available nitroxide spin labels, most often the methanethiosulfonate spin label (MTSL) are investigated.^29^ The accessible distance range for samples in deuterated solutions is frequently given as 1.5–8 nm.^30^ Distances up to 16 nm have been demonstrated exploiting protein deuteration also referred to as perdeuteration.^31,32^

Biological systems hugely benefiting from a more routine application of long-range PELDOR include chaperonins,^31^ protein RNA complexes such as the ribosome,^33^ viral capsids^34^ and large molecular machineries such as secretion systems.^35^ For such large biomolecules PDS can be very powerful in disentangling conformer distributions that are difficult to access by crystallography or cryogenic electron microscopy (cryo-EM).

The PELDOR distance limitations arise from the need to resolve the distance dependent dipolar coupling between two spins (eq 1), on the one hand and coherence lifetime on the other.

1 $$\omega_{12}\left(\theta ,r\right)\propto \frac{1}{r_{12}^{3}}$$

With increasing *r*_12_^3^ the dipolar oscillation frequency ω_12_(*θ,r*) will decrease and the oscillation period will increase. Taking for example a distance of 10 nm between the unpaired electrons, primary data of 19.3 and 38.5 μs will be needed to reliably extract the mean distance or the mean distance and distribution width (Supporting Information (SI), section 2.5).^36^ However, the length is limited by the signal-to-noise ratio (SNR, SI section 2.8) mainly depending on transverse dephasing (characterized by the phase memory time *T*_m_). *T*_*m*_ can be shortened by local spin concentration through instantaneous diffusion effects,^37^ by measurement temperatures allowing local dynamics and by a high number of nearby nuclear spins. However, most experiments are performed at low micromolar concentrations and helium temperatures^38,39^ in deuterated buffer^40^ where all these effects are largely mitigated. Perdeuteration can extend *T*_m_ even further.^31,41^

Consequently, measurements of distances exceeding 8 nm are sparse in literature.^32,42,43^ Even rarer are measurements above 10 nm which usually require perdeuteration, as demonstrated for oligonucleotides^44^ and proteins.^31,45^ To the best of our knowledge these are the only three reported measurements of samples with average distances over or close to 10 nm, highlighting the importance of investigating the distance range above 10 nm systematically with the available set of methods.

Here, the dimeric antiparallel coiled coil domain of the TRIM25 ^7,47,48^ (Figure 1a) protein was investigated over a wide range of distances and conditions.

The full length TRIM25 protein was truncated to cover only its antiparallel coiled coil domain within residues 189–379 and the native cysteine was removed (see SI, for further information about construct/mutant design, expression, purification and characterization). To cover a wide variety of distances several cysteine mutations were inserted, based on the expected distances between the residues (Figure 1b,c). For details on modeling approaches and software see SI, section 1.9.^46^

TRIM25 mutants in the distance range between 8 and 13.5 nm were prepared to test the common condition of nondeuterated proteins in deuterated buffer and cryoprotectant. Respective samples were subjected to PDS (SI, section 1.8.3, Figure 2). Whereas dipolar modulation is obvious for the shortest distance mutant (D221R1 Figure 2a, magenta trace) resolving between background and modulation becomes exceedingly difficult making data analysis challenging. While the two shortest distance mutants (D221R1, R218R1) were analyzable with automated workflows that avoid user bias^49,50^ (SI, section 1.8.4) this failed for longer distance mutants where manual distance extraction including user input on background models was required (see SI, section 1.8.4 for details).^51,52^ Introducing a compactness criterion^52^ to resolve ambiguity between high local concentrations and long-distance contributions mainly led to a decreased mean distance (SI, section 2.6). The maximum mean distance extractable with this approach was about 10.5 nm (N214R1, Figure 2a, green trace), which is significantly above the often-cited distance limit of 8 nm. The TRIM25 coiled-coil domain represents a minimalistic scaffold for distance measurements and thus fewer protons in proximity of the spin label than would be the case in the average globular protein spanning 18 nm and beyond. For mutants with even longer distances (S211R1 and T207R1, Figure 2a and b, violet and cyan trace) distance analysis failed.

**Figure 2:**
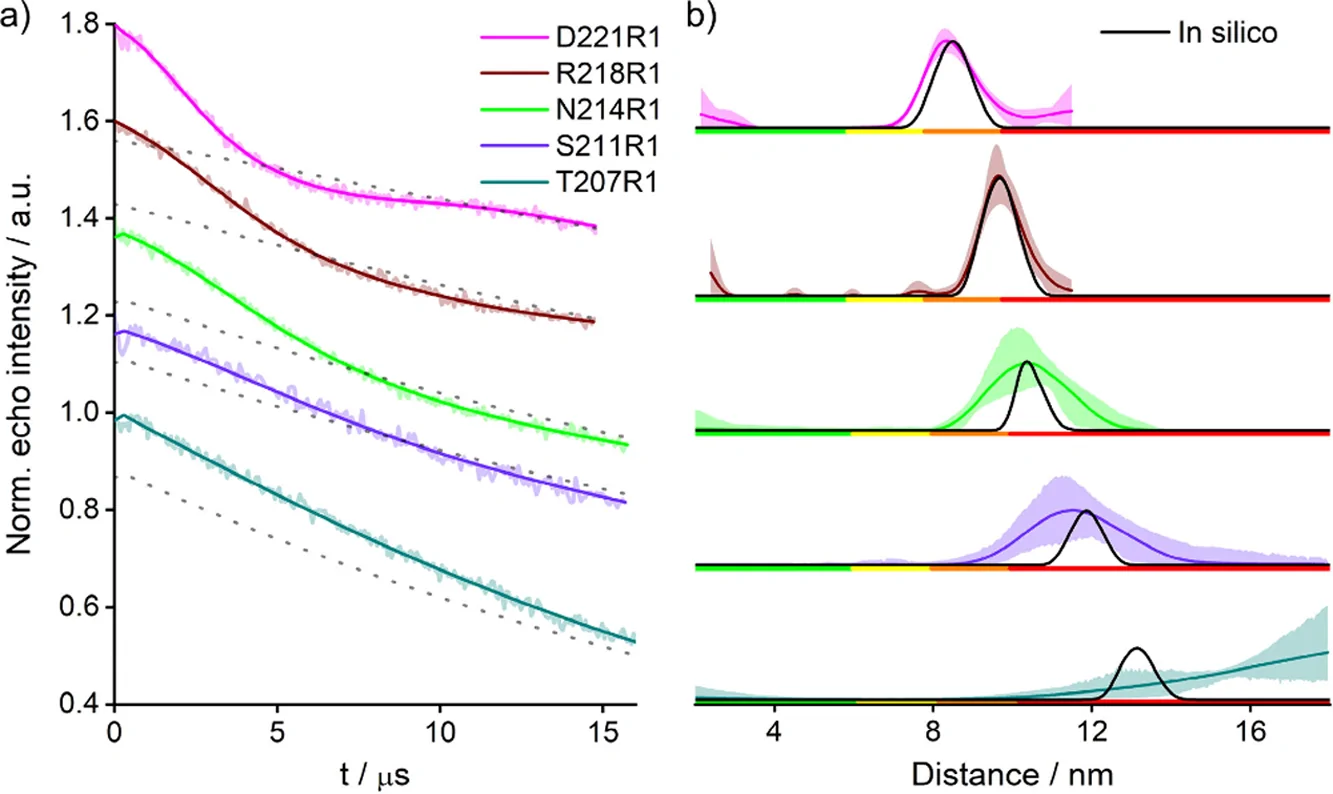
PELDOR measurements of TRIM25 in deuterated buffer at 50 K from the shortest D221C (magenta), R218C (brown), N214C (green), S211C (violet) to the longest T207C (dark cyan) mutant. (a) Time traces in the respective color code with the fitted background (black, dot) and (b) experimental distance distributions. The predicted distance distributions from ChiLife (black), normalized to the maximum of the experimental distance distribution and the reliability of the distance distributions is depicted by the color bars from green to red.

5-pulse DEER^53^ (SI, section 1.8.3) has allowed extending data acquisition windows by a factor of 1.4 in deuterated solutions as described in the literature.^53^ Measurements (SI, section 2.7) with longer primary data but also featuring characteristic unwanted dipolar pathways^54^ were recorded. The resulting distance distributions varied depending on whether the compactness criterion was applied (SI, section 2.7). Fitting to 4-pulse and 5-pulse DEER traces globally might stabilize the resulting fits but this was beyond the scope of this work. Here, we decided to explore extending *T*_*m*_ by perdeuteration.

To achieve the longest possible phase memory times *T*_m_ near quantitative deuteration of the protein is required.^37,38^ In case of TRIM25 mass spectrometry data indicated quantitative deuteration (SI, section 2.2). This led to a ∼5–8 fold increase in *T*_*m*_ which should translate into PDS traces with >100 μs length becoming accessible. Here, some common measurement parameters need to be revisited. At these extended acquisition windows detrimental effects from sample concentration and temperature dependent echo dephasing amplify massively. Thus, for 100 μs, 40 K and 40 μM spin concentration, is the calculated optimum (SI, sections 2.8 and 2.9). For long time windows the difference between measurement temperatures amplifies. For 10 μs dipolar evolution, a measurement at 80 K has still half the sensitivity of a measurement at 50 K, while enabling cooling with liquid nitrogen rather than more costly liquid helium. Sensitivity losses can be counteracted with novel labels that improve *T*_*m*_ especially at high temperatures.^55^ However, for the case of MTSL and very long distances with long dipolar evolution (i.e., 100 μs) 40 K is more than 10-fold more sensitive than 80 K. To further improve sensitivity the dipolar evolution step width was increased to 1 μs which still allows excellent sampling under conditions found here. The data acquired (Figure 3) enabled the unbiased extraction of distance distributions centered at up to 17.3 nm with automated workflows avoiding more complex analysis (and risk of user bias). The differences in background (Figure 3a) between the S190R1 and the other mutants are attributed to its concentration (30 μM protein dimer). A slight shift to shorter distances of the measured distributions compared to the simulated data (Figure 3b) was observed. This deviation of measured distance distributions from the crystal structure and structure predictions might be due to disparity in curvature between the solution and crystalline state. We also cannot, at this time, rule out dynamic heterogeneity as the cause, although the PELDOR data appears to show a remarkable rigidity in the coiled coil. The present solution data clearly shows a reduced mean distance, reliable distribution widths would be required to understand if this is caused by a more curved minimum energy structure or a distribution of different curvature degrees in solution.

**Figure 3:**
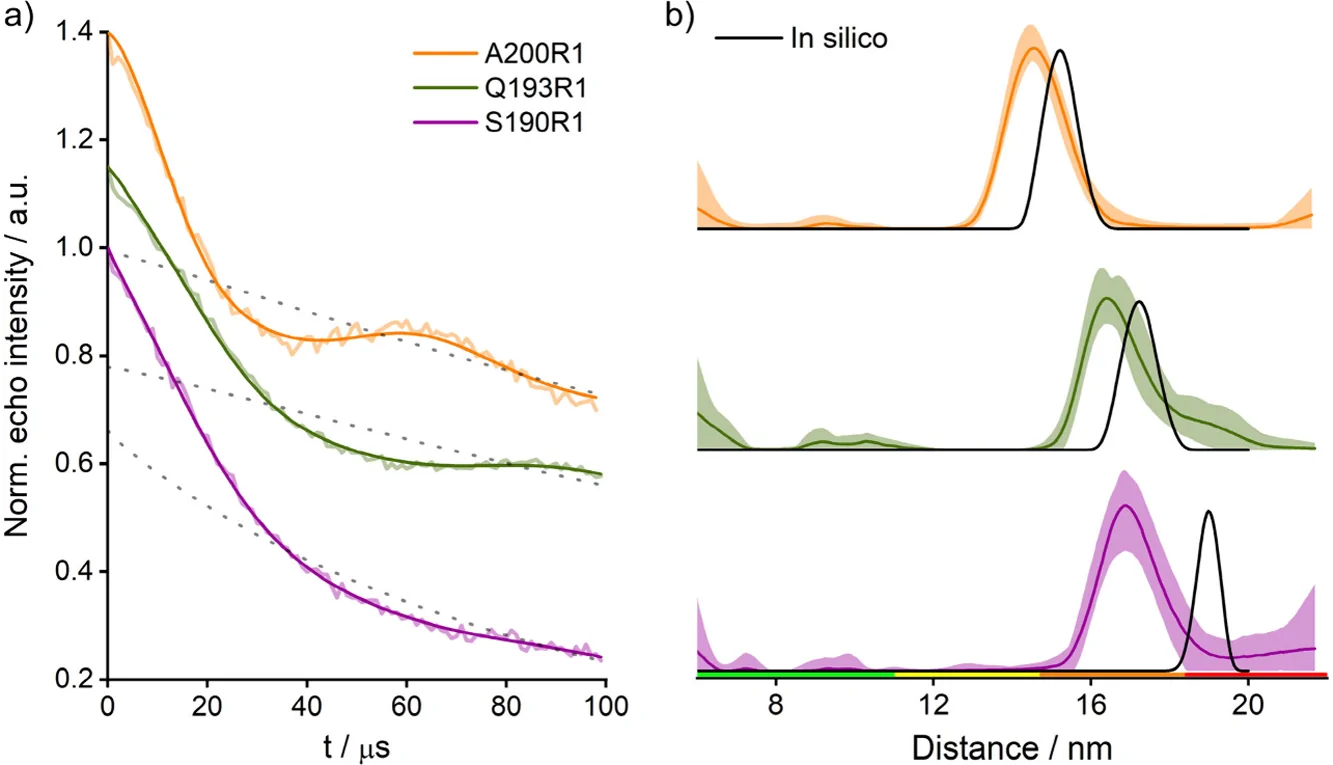
PELDOR measurements of perdeuterated TRIM25 at 40 K in deuterated buffer from the shortest A200R1 (orange), Q193R1 (olive), to the longest S190R1 (purple) distance mutant. (a) Time traces in the respective color code with the fitted background (black, dotted) and (b) the determined experimental distance distributions. The predicted distance distributions from ChiLife (black), normalized to the maximum of the experimental distance distribution and the reliability of the distance distributions is depicted by the color bar from green to red.

Additionally, PELDOR measurements via the 3-pulse or 5-pulse DEER sequence^26^ (SI, section 1.8.3) were investigated. The gain in time window length of the 5-pulse DEER experiment was negligible in the perdeuterated case (SI, section 2.7), whereas the Hahn echo exploited in the 3-pulse DEER measurement features a higher echo intensity than the refocused echo, which is used in the 4-pulse sequence. This increases the accessible distance range further (SI, section 2.10).^56^ The common drawback is the inaccessible zero-time giving rise to increased uncertainties for short distances and broad distributions during subsequent analysis. In the present case these effects are negligible as confirmed by our results and sensitivity increases between 20 and 50% were observed (SI, section 2.10).

All the acquired data exhibits an SNR > 20 in less than 24 h (SI, section 2.10), leaving room to extend the measurement time further, potentially extending the measurable trace length and thereby the accessible distance range. Therefore, the Q193R1 mutant, featuring the best SNR, was used for investigating the maximum accessible trace length in ∼72 h measurement time (SI, section 2.11). The resulting data yielded SNRs close to 10 at dipolar evolution times of 150 and 155 μs, respectively, which is equivalent to almost a 2-fold increase in comparison to the previously published longest trace recorded.^31^ When back-calculating the average distance that should be reliably extractable 20 nm should be within reach.

To summarize, we have shown that PELDOR measurements under typical sample conditions without protein deuteration are feasible up to 10.5 nm. Going to even longer distances already the interpretation of the mean distance becomes unreliable. Moving to PELDOR with a 5-pulse DEER sequence, average distances beyond 10.5 nm are accessible but the data modeling becomes more challenging and potentially less robust.

As reported previously^31,32^ perdeuteration enhanced *T*_m_ by 5 to 8-fold facilitated by almost complete proton-to-deuteron exchange. The SNR and thereby the maximal accessible distance range for these measurements were considerably enhanced by optimizing parameters for dipolar evolution times approaching 150 μs—effectively double the longest reported dipolar evolution times in literature.^31^ Here, the application of the 3-pulse DEER experiment is highly beneficial since further improvements of the signal can be achieved without major effects from the inaccessible zero-time. In this study, 5-pulse DEER did not lead to further gains for perdeuterated samples.

The longest measured mean distance of 17.3 nm is well beyond literature precedent, and our data predicts that 20 nm is within reach. Furthermore, TRIM25 featured a higher degree of curvature in solution than suggested by the crystal structure and structure prediction. All these results were obtained adhering to the most recent community guidelines for acquisition, analysis and interpretation of PELDOR/DEER data using unbiased analysis workflows.^30^

This study opens access to length scales typical for large protein assemblies and molecular machines and might enable valuable PELDOR contributions to integrated studies on complex biological systems. Thus, providing robust long-range restraints that complement high-resolution but locally focused techniques such as crystallography, NMR, and cryo-EM, will enable more detailed modeling of large biomolecular systems and their conformational space. The research data underpinning this publication will be accessible at DOI: https://doi.org/10.17630/4e23626f-407a-4848-a62c-f8689956761d.^62^

## Supplementary Material



## Notes

Detailed experimental procedures, TRIM25 purification and characterization, comparison of data analysis procedures, 3-pulse and 5-pulse DEER data.^57–62^ (PDF)
