# Supplementary material for: Spins Afar—Long-Range Pulse Dipolar EPR Spectroscopy Uncovers Curvature in TRIM25’s Coiled Coil
Source: J Phys Chem Lett. 2026 Oct 5;17(41):11915–20. doi: 10.1021/acs.jpclett.6c02671 (PMC13679085; doi:10.1021/acs.jpclett.6c02671)
Supplement: Supplementary file 1 [file jz6c02671_si_001.pdf]

Spins Afar - Long-Range Pulse Dipolar EPR Spectroscopy Uncovers Curvature in TRIM25's Coiled Coil

*Yannik Limbach[a,b], Katrin Ackermann[a], Aisika Chakraborty,[a] David G. Norman[c], Bela E. Bode[a]**

[a]EaStCHEM School of Chemistry, Biomedical Sciences Research Complex and Centre of Magnetic Resonance, University of St. Andrews, North Haugh, St Andrews KY16 9ST, Scotland, UK

[b]Clausius-Institute for Physical and Theoretical Chemistry, University of Bonn, Wegelerstr. 12, 53115 Bonn, Germany

[c]School of Life Sciences, University of Dundee, Medical Sciences Institute, Nethergate Dundee DD1 4HN, Scotland, UK

AUTHOR INFORMATION

Corresponding Author

*beb2@st-andrews.ac.uk

Table of Contents

1. Methods 3
1.1 Construct/Mutant design 3
1.2 Protein expression and purification 3
1.3 Perdeuterated expression and purification 4
1.4 MTSL labeling 5
1.5 Mass spectrometry 5
1.6 EPR sample preparation 5
1.7 CW-EPR experiments 6
1.7.1 Instrumentation and measurement parameter 6
1.8 Pulse EPR experiments 6
1.8.1 Instrumentation 6
1.8.2 Relaxation time measurement 7
1.8.3 PELDOR experiments 8
1.8.4 PELDOR data processing and analysis 10
1.9 Modeling 10
2. Results and discussion 10
2.1 Chromatograms and SDS-PAGE gel electrophoresis 10
2.2 Mass spectrometry 14
2.3 CW-EPR spectroscopy 19
2.4 Influence of the cryoprotectant on T_m 20
2.5 Quantitative description of trace length distance ratio 20
2.6 DeerLab analysis, comparison between compactness and non-compactness 21
2.7 5p DEER measurements 23
2.8 Relaxation time measurements and optimal SNR for perdeuterated samples 25
2.9 Concentration considerations 26
2.10 3p/4p DEER comparison 28
2.11 Maximal accessible trace length 3p/4p DEER 29
3. Literature 30

1. Methods

1.1 Construct/Mutant design

For this study, a sub-domain of the tripartite motif protein 25 (TRIM25) from *Homo Sapiens* encoding a dimeric antiparallel coiled coil was designed. Therefore, the full length protein (Uniprot ID: Q14258) was truncated to include only residues 189-379 as previously described by Sanchez *et al*.[1] In order to facilitate protein purification an N-Terminal His6-tagged SUMO leader sequence was added. The amino acid encoding nucleotide sequence was inserted into a pET28a(+) vector for protein expression in *E. coli* bacteria. The native cysteine in the protein was removed *via* QuickChange mutagenesis,[2] this initial construct will be referred to as TRIM25 here and thereafter. New cysteines were inserted into this plasmid in positions 221, 218, 214, 211, 207, 200, 193 and 190. The respective primer pairs are listed in Table S1.

Table S1: Primer pairs for the removal of the native cysteine and introduction of labeling positions.

Primer	Sequence
C351S fwd.	5'-TGAAGCAGTCCATCGGCCGTCTGCAAGAGCCGA-3'
C351S rev.	5'-CCGATGGACTGCTTCAATTCGTTCTTGAGATCAATGGTG-3'
D221C fwd.	5'-CGCTGTGCGACGTTCGTAACCGCCAGCAGGATGTG-3'
D221C rev.	5'-GAACGTCGCACAGCGCACGAGAGGCACCGTTGATC-3'
R218C fwd.	5'-GTGCCTCTTGTGCGCTGGACGACGTTCGTAACCG-3'
R218C rev.	5'-GCGCACAAGAGGCACCGTTGATCTGGCTATACATTACG-3'
N214C fwd.	5'-CAGATCTGCGGTGCCTCTCGTGCGCTGGACGACGT-3'
N214C rev.	5'-AGGCACCGCAGATCTGGCTATACATTACGGTCAGCTTGTGG-3'
S211C fwd.	5'-CGTAATGTATTGCCAGATCAACGGTGCCTCTCGTGCG-3'
S211C rev.	5'-GATCTGGCAATACATTACGGTCAGCTTGTGGCGCAGG-3'
T207C fwd.	5'-CCACAAGCTGTGTGTTATGTATTCCCAGATCAACGGC-3'
T207C rev.	5'-ATAACACACAGCTTGTGGCGCAGGGTAGCCTCCA-3'
A200C fwd.	5'-CTTGGAATGTACCCTGCGCCACAAGCTGACCGTAATGTATA-3'
A200C rev.	5'-GCAGGGTACATTCCAAGTCTGCGCTCGCTTGAGACAG-3'
Q193C fwd.	5'-TGAGCTGTGCGAGCGCGGACCTGGAGGCTACC-3'
Q193C rev.	5'-CGCTCGCACAGCTCAGGCTCGCACCGCCGATT-3'
S190C fwd.	5'-GGTGCGTGCCTGTCTCAAGCGAGCGCAGACTTGG-3'
S190C rev.	5'-AGACAGGCACGCACCGCCGATTTGTTCTCTATGCGCTT-3'

The modified plasmid was afterwards transformed into DH5α competent *E. coli* cells (NEB), multiplied and extracted from the bacteria and checked for successful insertion of the mutation *via* sequencing.

1.2 Protein expression and purification

Expression and purification of the respective TRIM25 mutants was adapted from Sanchez *et. al*.[1], with modifications to the procedure. The plasmid of the respective mutants was transformed into BL21 (DE3) competent *E. coli* cells. A single colony from an agar plate

(kanamycin resistance) was picked and grown overnight at 37 °C and 180 rpm in 50 mL Luria-Bertani (LB) medium supplemented with 50 µg/mL kanamycin. The next day 1 L main culture was prepared by adding 20 mL of preculture to each liter of LB medium as well as 50 µg/ml kanamycin. The cultures were then incubated at 37 °C and 180 rpm until an OD_{600} ~ 0.6-0.8 was reached. Protein expression was induced by the addition of 0.2 mM isopropyl-β-D-1-galactopyranoside (IPTG) and cultures were left shaking overnight at 16 °C. To pellet the bacteria, cultures were centrifuged for 20 min, 4 °C and 4000 rcf.

The resulting pellet was resuspended in 5 × v/w wash buffer (50 mM Tris Base, 300 mM NaCl, 20 mM imidazole, pH 8) and 3 mM dithiothreitol (DTT) were added. Cells were then lysed *via* sonication (10 cycles, 45% amplitude, 30 s on/30 s off). The solution was centrifuged once more for 30 min, 4 °C and 45,000 rcf. The supernatant was filtered (0.2/0.8 µm membrane filter) before loading on a 5 mL HisTrap HP (Cytiva) column equilibrated with wash buffer. Elution of the protein was achieved by gradually increasing the imidazole concentration with elution buffer (50 mM Tris base, 300 mM NaCl, 300 mM imidazole, pH 8). The protein content of the resulting fractions was checked with SDS-Page and suitable fractions were pooled. To remove the solubility tag from the protein, the solution was dialyzed overnight into PBS Buffer with addition of 1:200 v/v Ulp1 protease (15 µM stock in 50% glycerol) and 3 mM DTT.

The next day the dialyzed solution was filtered once more before loading onto the equilibrated HisTrap HP column. Since the His-Tag was cleaved, the protein should be in the wash fraction of the column, which was validated with SDS-PAGE. The respective fractions were pooled and concentrated with a centrifugal concentrator with 10 kDa molecular weight cut-off (MWCO, Pall) until the volume reached approximately 1 mL. In a final purification step the solution was filtered once more with a syringe filter (0.2 µm membrane filter) and loaded onto a HiLoad 26/600 200 pg (Cytiva) size exclusion column (SEC) which has been equilibrated with SEC buffer (30 mM Tris base, 100 mM NaCl, pH 8). The resulting fractions were evaluated for their protein content with SDS-PAGE and the protein containing fractions were pooled and concentrated with a centrifugal concentrator MWCO 10 kDa (Pall) until a sufficient concentration was reached. The purity of the desired protein was evaluated with electrospray ionization (ESI) mass spectrometry, and the solution was then flash frozen in liquid nitrogen and stored for further use at -80 °C.

1.3 Perdeuterated expression and purification

Perdeuterated TRIM25 mutants were expressed based on the previously presented procedure with modifications for deuterated expression adapted from Schmidt *et al.*[3]

As stated above, a single colony from an agar plate (kanamycin resistance) was picked and grown overnight at 37 °C and 180 rpm in 5 mL LB medium supplemented with 50 µg/mL kanamycin. The next day 105 mL deuterated M9 minimal medium was prepared supplemented with 50 µg/mL kanamycin, 0.5 g/L D-ISOGRO (Merck) and 2 g/L d12-glucose (Merck). The LB culture was centrifuged for 5 min at 4000 rpm. The supernatant was then

discarded, and the pellet was washed with 5 mL deuterated M9 medium. After another centrifugation step the resulting pellet was redissolved in deuterated M9 medium and transferred into the remaining 100 mL medium. The cultures were then incubated at 37 °C and 130 rpm until an OD_{600} ~ 0.8 was reached. Protein expression was induced by the addition of 0.2 mM IPTG and cultures were left shaking overnight at 25 °C. To pellet the bacteria, cultures were centrifuged for 10 min, 4 °C and 4000 rpm.

The purification procedure was carried out with minor modifications, since the protein amount was greatly reduced due to lower expression levels and smaller main cultures the final SEC step was omitted. The protein purity after the 2nd HisTrap HP column was sufficiently high as presented in the results section.

1.4 MTSL labeling

The spin labeling with the (2,2,5,5-tetramethylpyrroline-1-oxyl-3-methyl) methanethiosulfonate (MTSL) label (Santa Cruz Biotechnology) was carried out as described previously[4] with minor modifications for TRIM25. Therefore, up to 50 nmol of the respective protein dimer were incubated in 1 mL SEC buffer (30 mM Tris base, 100 mM NaCl, pH 8) with the addition of 3 mM DTT. The mixture was then stored on ice for 2 h before removal of the reducing agent *via* a PD10 column (Cytiva). A 10-fold molar excess per cysteine of a 100 mM stock of MTSL in DMSO was added to the protein containing fraction and incubated overnight at 4 °C in the dark. The next day, the solution was passed over a PD10 column in multiple fractions, the protein-containing eluate was concentrated using a centrifugal concentrator with a 10 kDa MWCO cutoff (Pall). Depending on the purpose the labeled protein was either freeze-dried to exchange the buffer into deuterated solvent for pulse dipolar EPR spectroscopy (PDS) or flash frozen in liquid nitrogen and stored at -80 °C. The labeling efficiency was assessed by ESI mass spectrometry and the absence of free label was confirmed *via* CW-EPR.

1.5 Mass spectrometry

ESI mass spectrometry was performed on samples before and after spin labeling. Samples were diluted to 1 µM in 1% formic acid (FA). 20 pmol per sample were injected onto the liquid chromatography (LC) system with a time of flight (TOF) mass spectrometer (MS) (Waters Xevo G2 TOF MS with Acquity HPLC) using a MassPrep cartridge column (Waters), applying a 5 min gradient from 95% water, 5% acetonitrile to 5% water, 95% acetonitrile (eluents supplemented with 1% FA). Data was collected in positive mode from 500-2500 *m*/*z*, and charged ion series deconvolution to 0.1 Da resolution was performed using the MaxEnt I algorithm utilizing a peak width at half height of 0.5 *m*/*z*.

1.6 EPR sample preparation

Continuous wave (CW) EPR samples were prepared by filling 20 µL of labeled protein solution into 20 µL glass capillaries (Brand) which were sealed by wax afterwards.

For the preparation of pulse EPR samples, freeze dried protein was redissolved in equal amounts of D_2O and the concentration was remeasured. The final protein concentration in the samples (Table S2) was adjusted by diluting with deuterated SEC Buffer and in the last step by mixing the solution 1:1 with d6-ethylene glycol (Sigma-Aldrich). Of this solution 65 µL were then transferred into a 3 mm (outer diameter) quartz tube (Baumbach) *via* Hamilton tubing and the sample was flash frozen in liquid nitrogen. In case of the N214R1 mutant a second sample with 1:1 d8-glycerol (Sigma-Aldrich) was prepared.

Table S2: Concentration of the protein in the respective pulse EPR samples for each mutant.

Mutant	c(Dimer) / µM	c(Monomer) / µM
D221R1	50	100
R218R1	50	100
N214R1	50	100
S211R1	50	100
T207R1	50	100
A200R1	20	40
Q193R1	20	40
S190R1	30	60

For the perdeuterated samples the concentration was reduced based on the calculations carried out in section 2.9.

1.7 CW-EPR experiments

1.7.1 Instrumentation and measurement parameter

All CW-EPR measurements were performed using a Bruker EMX 10/12 spectrometer equipped with an ELEXSYS Super Hi-Q resonator, at X-band frequencies (9.9 GHz) and at 298 K with a 100 kHz modulation frequency.

Samples were recorded using a 120 G field sweep centered at 3505 G, a time constant of 20.48 ms, a conversion time of 18.67 ms, and 1714 points resolution. An attenuation of 20.0 dB (2 mW power) and a modulation amplitude of 0.7 G were used. To calculate a spin count, spectra were phase and background corrected and then in two individual steps integrated in the Xenon software (Bruker). The resulting double integral was then compared to a 100 µM solution of 4-Hydroxy-2,2,6,6-tetramethylpiperidine-1-oxyl (4-Hydroxy-TEMPO; Acros) as a standard.

1.8 Pulse EPR experiments

1.8.1 Instrumentation

Pulse EPR experiments were performed using a Bruker ELEXSYS 580 pulse EPR spectrometer. Temperatures were maintained using a cryogen-free variable temperature cryostat (Cryogenic Ltd.) operating in the 1.8-300 K temperature range. All measurements were performed at 50

or 40 K, using a high-power 150 W travelling-wave tube (TWT; Applied Systems Engineering) at Q-band (34 GHz) in an overcoupled 3 mm cylindrical resonator (Bruker ER 5106QT-2w).

1.8.2 Relaxation time measurement

To determine the T_1 and T_m relaxation and decoherence times, inversion recovery and 2 pulse (2p) electron spin echo decay experiments were conducted. To assess the influence of the measurement temperature experiments were conducted from 20 K to 80 K in 10 K steps. The utilized pulse sequences are depicted in Figure S1 with the respective parameters in Table S3. To extract the resulting relaxation times mono exponential decays were fitted to the data,

$$f(T) = a \cdot \exp\left(\frac{-T}{T_1}\right) + c \quad (1)$$

$$f(2\tau) = a \cdot \exp\left(\frac{-2\tau}{T_m}\right) + c \quad (2)$$

with a and c normalization factors. The inversion recovery measurements were multiplied by -1 to allow the fitting of equation (1) to the data. In general, fitting these types of experiments with stretched exponential decays in case of the 2p echo decay experiments and bi-exponentials for the inversion recovery has been shown to give more accurate values for the respective relaxation times.[5] For concentration optimization tests and assessment of accessible time windows in 4p and 5p DEER measurements refocused echo decay experiments (RED) were conducted (Figure S1c).

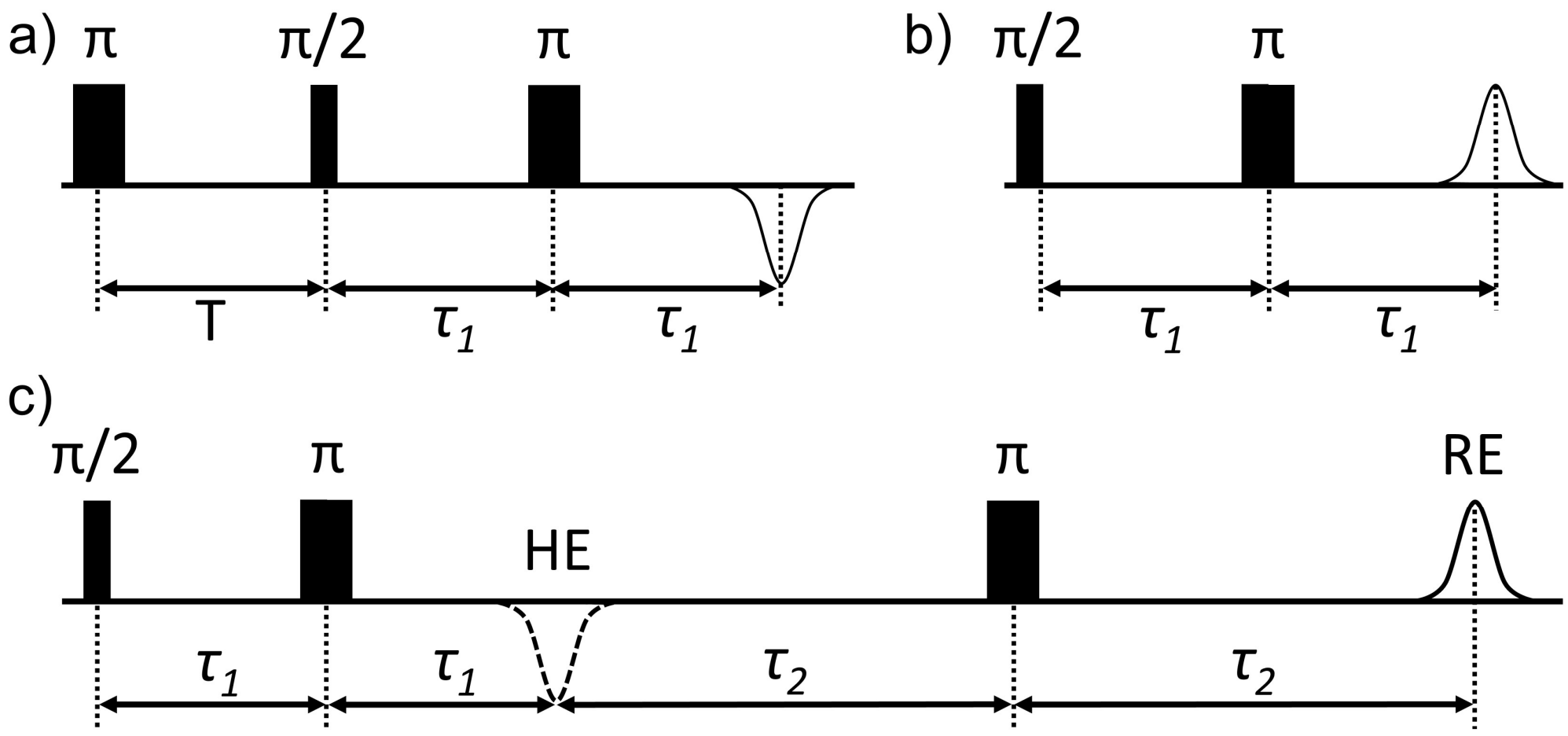

Figure S1: Pulse sequences of the a) Inversion recovery experiment, b) 2p echo decay experiment and c) Refocused echo decay (RED) experiment.

Table S3: Parameters for a) RED, 2p echo decay and b) Inversion recovery experiment.

a)

Variable	RED	2p echo decay
$(\pi/2)$	16 ns	16 ns
(π)	32 ns	32 ns
τ_1	760 ns	400 ns
$\tau_{2,\ increment}$ / $\tau_{1,\ increment}$	128 ns	128 ns
Shot Repetition Time (SRT)	5.9 ms	80 ms (20 K) – 3 ms (80 K)

b)

Variable	Inversion recovery
$(\pi/2)$	16 ns
(π)	32 ns
$(\pi)_{inv.}$	32 ns
τ_1	800 ns
T_{start}	1000 ns
$T_{increment}$ / $\tau_{1,\ increment}$	2.4 ms (20 K) – 40 µs (80 K)
Shot Repetition Time (SRT)	600 ms (20 K) – 20 ms (80 K)

1.8.3 PELDOR experiments

Four pulse pulsed electron-electron double resonance (PELDOR) experiments were performed using the double electron–electron resonance (DEER) sequences depicted below (Figure S2) using a 2-step phase cycle and 8-step τ_1/τ_2 modulation averaging with 16 ns steps. Measurements were conducted at 50 K or 40 K (perdeuterated) using the parameters listed below (Table S4). The detection frequency was applied with a frequency offset of -100 MHz with respect to the pump frequency. The pump pulse length was adjusted by a transient nutation experiment. All pulses in this scheme are rectangular and were set up *via* ELDOR or MPFU channels, respectively. Three pulse DEER experiments were set up in the same way with a deadtime delay $t_{initial}$ of 500 ns and without modulation averaging.

Five pulse DEER measurements were performed using the pulse sequence depicted below (Figure S2c) using a 16-step phase cycle and 8-step τ_1/τ_2 modulation averaging with 16 ns length. Measurements were conducted at 50 K using the parameters listed below (Table S5). The detection frequency was applied with a frequency offset of -100 MHz with respect to the pump frequency. This experiment was driven *via* the AWG 2 (Bruker). The detection pulses at frequency ν_a were Gaussians and as pump pulse a hyperbolic secant pulse HS{1,6} with the parameters in Table S5 was used.

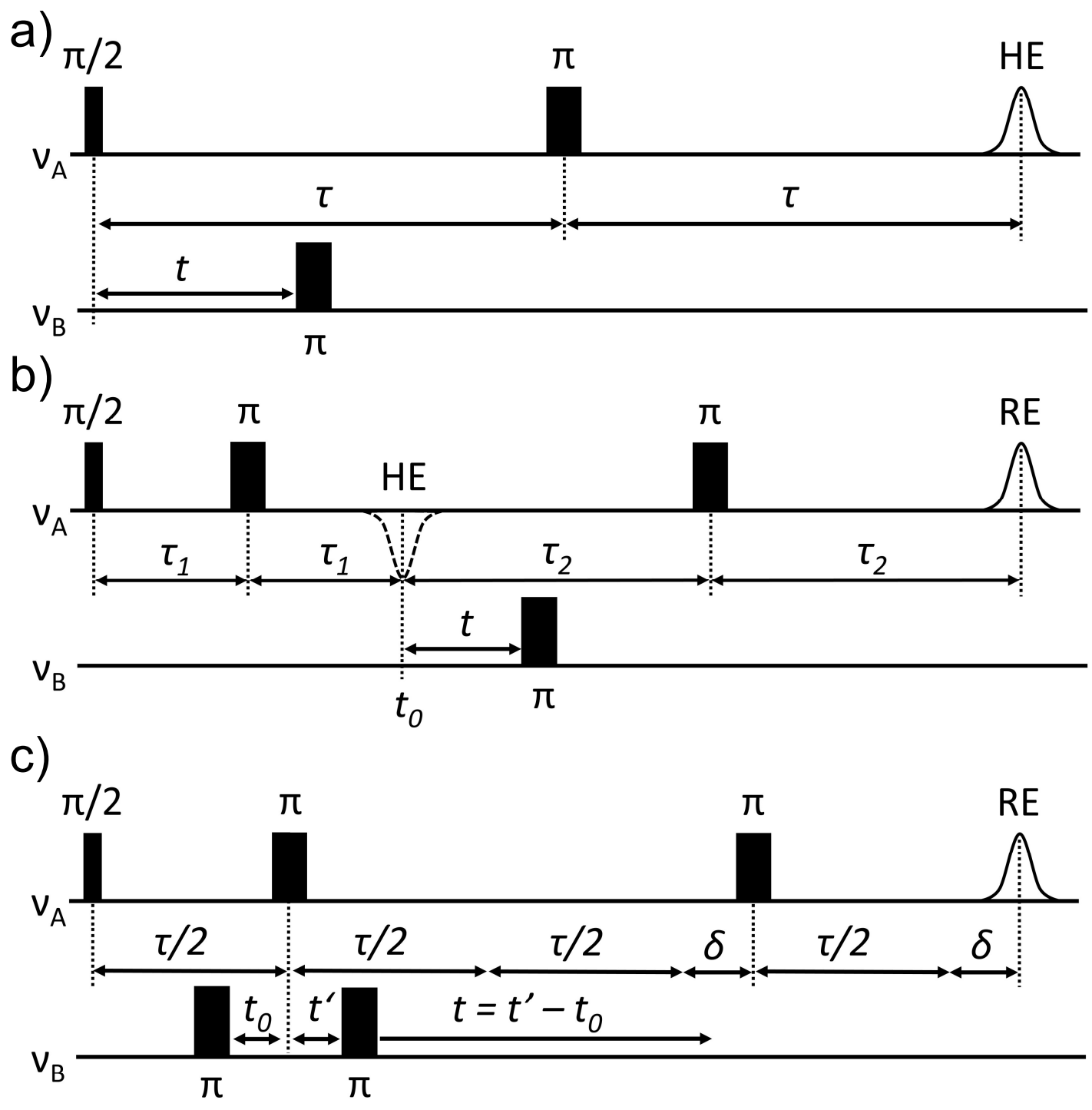

Figure S2: PELDOR experiments, a) 3p, b) 4p and c) 5p DEER sequences.

Table S4: Parameters for 3 pulse DEER and 4 pulse DEER measurements. The shot repetition time is abbreviated with SRT.

Variable	3 pulse	4 pulse
$(\pi/2)_A$	16 ns	16 ns
$(\pi)_A$	32 ns	32 ns
$(\pi)_B$	10 ns	10 ns
$t_{initial}$ / τ_1	500 ns	760 ns
τ / τ_2	Depending on mutant	Depending on mutant
SRT (normal)	3 ms	3 ms
SRT (deuterated Prot.)	5.9 ms	5.9 ms

Table S5: Parameters for 5 pulse DEER and the utilized AWG pump pulse parameters. The shot repetition time is abbreviated with SRT.

Variable	5 pulse
$(\pi/2)_A$	24 ns
$(\pi)_A$	48 ns
τ_1	760
τ_2	Depending on mutant
SRT	1.47 ms
$(\pi)_B$ length	150 ns
$(\pi)_B$ width	70 MHz
$(\pi)_B$ offset	85 MHz

1.8.4 PELDOR data processing and analysis

Four and three pulse DEER data was analyzed using the ComparativeDEERAnalyzer2.0 (CDA2.0) in DeerAnalysis2022[6]. If necessary, up to 5% of the trace were cut off at the end to remove artifacts. In all cases where CDA data is presented the consensus distribution and fitted traces are shown.

In cases where the CDA analysis failed, data was analyzed with the python package DeerLab.[7] The 4p DEER model with one pathway was used in combination with the compactness criterion[8] and without.

Five pulse DEER data was analyzed with DeerLab. A multi pathway analysis[9] of the five pulse experiments was performed in combination with the application of the compactness criterion. To increase the accuracy of DeerLab predictions, bootstrapping was performed with 100 iterations.

1.9 Modeling

The predicted distance distribution for the TRIM25 mutant labeled with MTSL was modelled based on a TRIM25 crystal structure (PDB:4LTB).[1] To predict the different distance distributions, the python software package ChiLife[10] was used in combination with the rotamer library "R1M".[11,12] Predictions were calculated with the rotamer library (RL), accessible volume (AV) and off-rotamer method (OR)[13] to account for different modelling options. In case of the conducted site scan in Figure 1b in the main text, the RL approach was used due to the significantly faster computation time. In all other cases the OR results are depicted in the main text.

2. Results and discussion

2.1 Chromatograms and SDS-PAGE gel electrophoresis

The purity of the resulting fractions in each of the chromatography steps carried out during the purification procedure was assessed *via* SDS-PAGE. The resulting gel images and chromatograms of the final SEC-steps are depicted in Figure S3-4. The first peak in each of the chromatograms was not added to the pool even though it was confirmed *via* mass spectrometry that this is also TRIM25, because these fractions contained minor protein impurities as visible in the SDS-PAGE gels.

In Figure S5 the perdeuterated samples are shown. Since the final SEC step was omitted for the perdeuterated samples the chromatogram of the second HisTrap will be shown alongside the respective gels. In all these purifications only the wash fraction of the column was pooled.

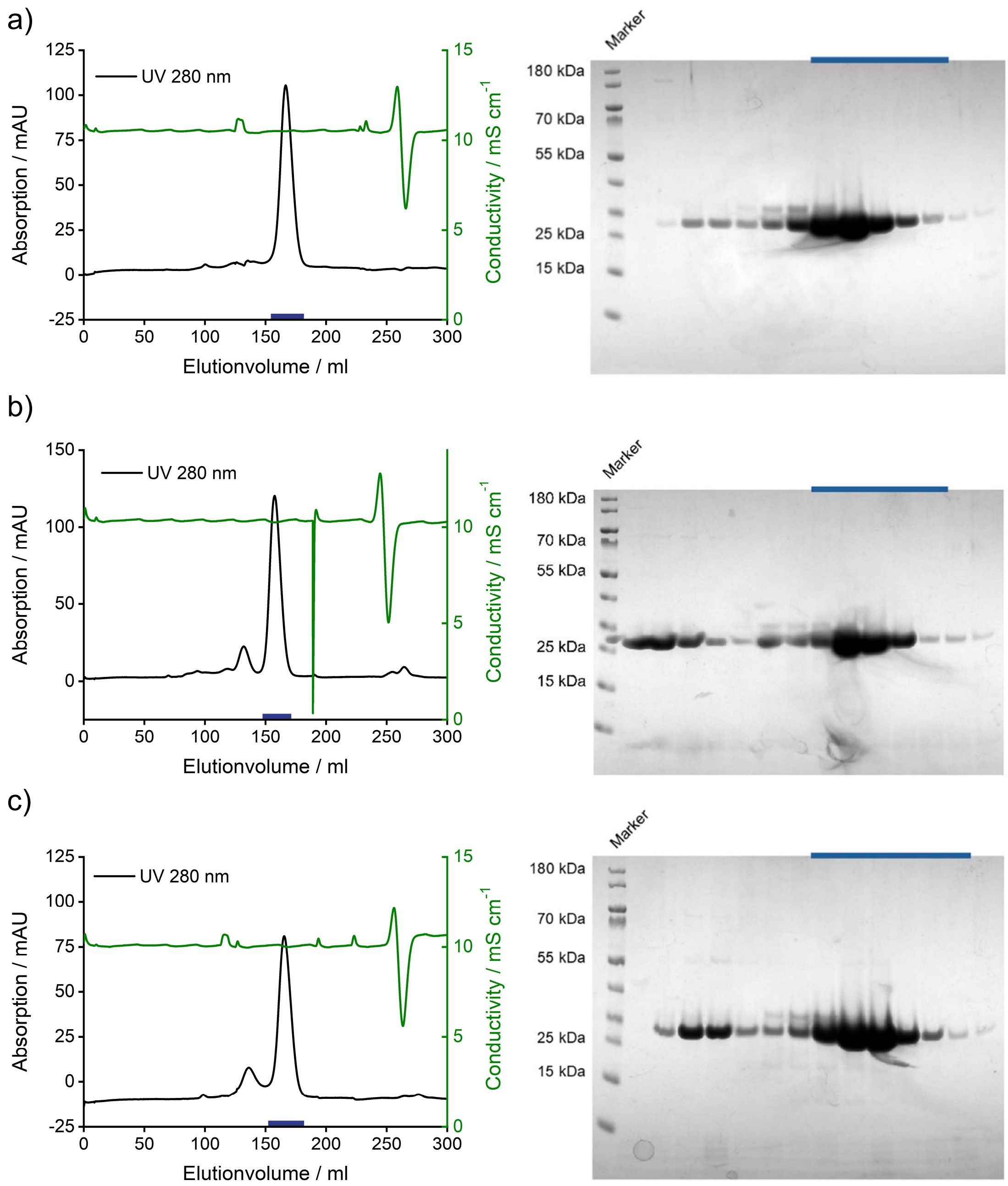

Figure S3: SEC chromatogram of the last purification step (left) and 4-12% Nu-PAGE gel image (right). The pooled fractions and their respective positions in the chromatogram are marked in blue. TRIM25 mutants, a) D221C, b) R218C and c) N214C.

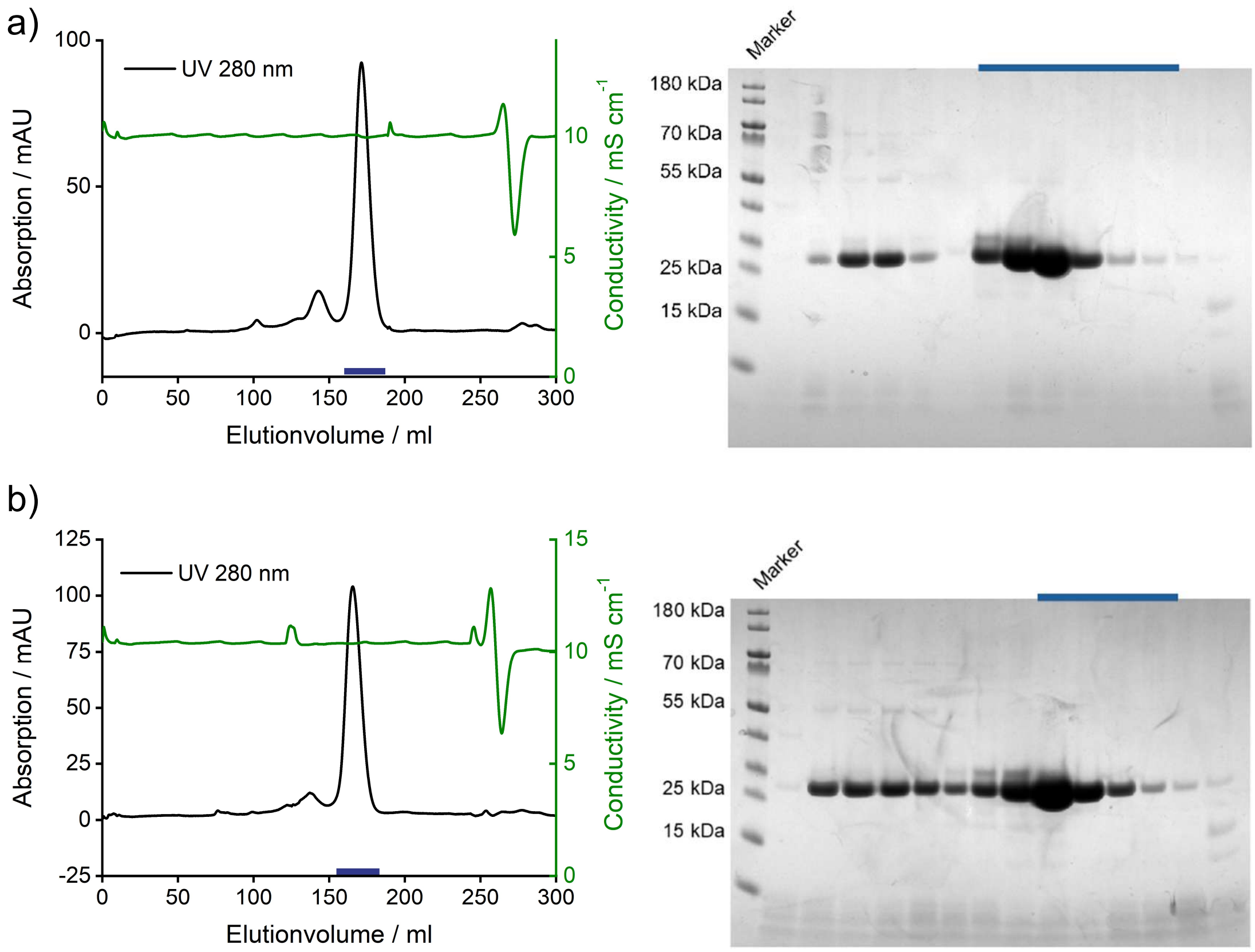

Figure S4: SEC chromatogram of the last purification step (left) and 4-12% Nu-PAGE gel image (right). The pooled fractions and their respective positions in the chromatogram are marked in blue. TRIM25 mutants, a) S211C and b) T207C.

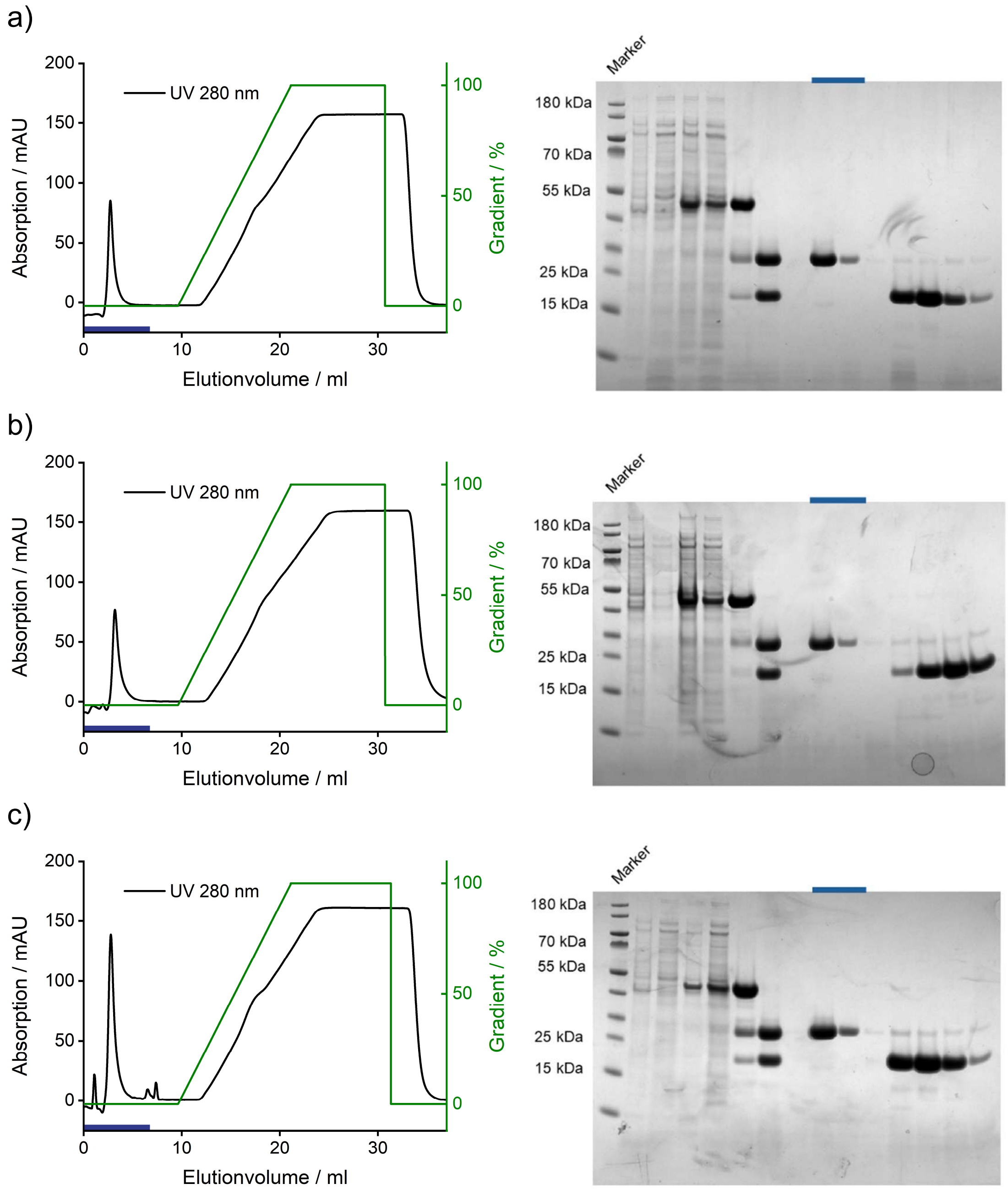

Figure S5: HisTrap column chromatogram after cleavage of the SUMO-Tag (left) and 4-12% Nu-PAGE gel image (right). The pooled fractions and their respective positions in the chromatogram are marked in blue. Perdeuterated TRIM25 mutants, a) A200C, b) Q193C and c) S190C.

2.2 Mass spectrometry

The resulting mass spectrometry data in Figure S6-S13 confirms quantitative labeling for all the prepared mutants and absence of any impurities. Masses found match the calculated ones within ±1 Da and in the perdeuterated samples ±2 Da as shown in

Table S6 and Table S7. The calculated masses for perdeuterated samples assume that all exchangeable protons are back exchanged to ^{1}H, since the labeling and sample preparation for mass spectrometry was executed in non-deuterated buffers. The number of exchangeable protons was calculated with the web application MSTools.[14]

Table S6: Calculated and measured masses for all non-perdeuterated samples with and without MTSL.

Mutant	Calculated mass / Da	Measured mass / Da
D221C	21790.68	21791.2
D221R1	21976.08	21975.7
R218C	21749.58	21750.5
R218R1	21934.98	21934.3
N214C	21791.67	21792.3
N214R1	21977.07	21976.7
S211C	21818.69	21818.3
S211R1	22004.09	22004.0
T207C	21804.66	21806.1
T207R1	21990.06	21989.5

Table S7: Calculated and measured masses for all perdeuterated samples with and without MTSL.

Mutant	Calculated mass / Da	Measured mass / Da
A200C	23010.69	23012.6
A200R1	23195.99	23197.0
Q193C	22952.64	22954.4
Q193R1	23137.94	23139.2
S190C	22995.69	22997.4
S190R1	23180.99	23182.6

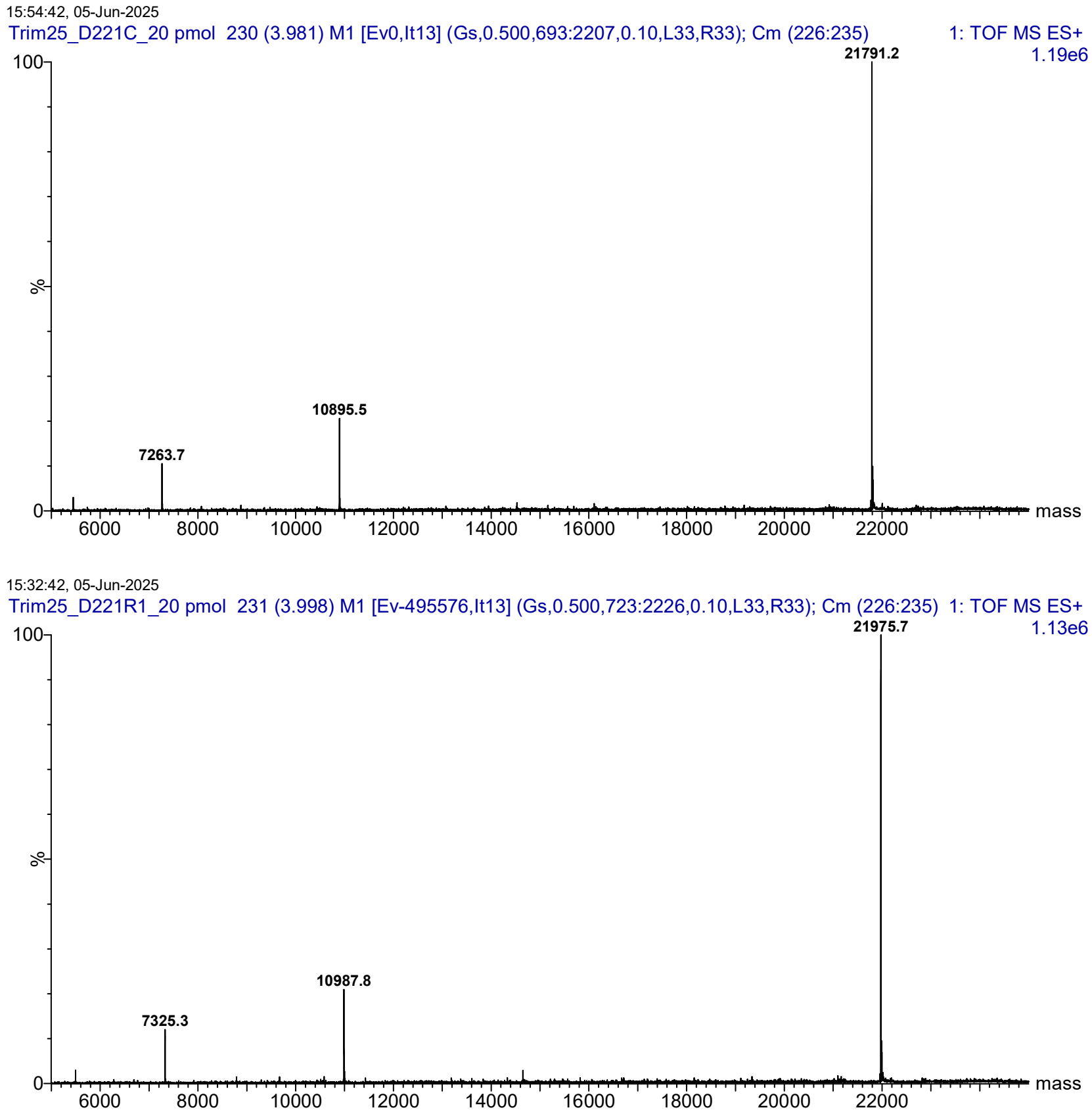

Figure S6: Deconvoluted ESI mass spectrometry data of TRIM25 D221C (top) and D221R1 (bottom).

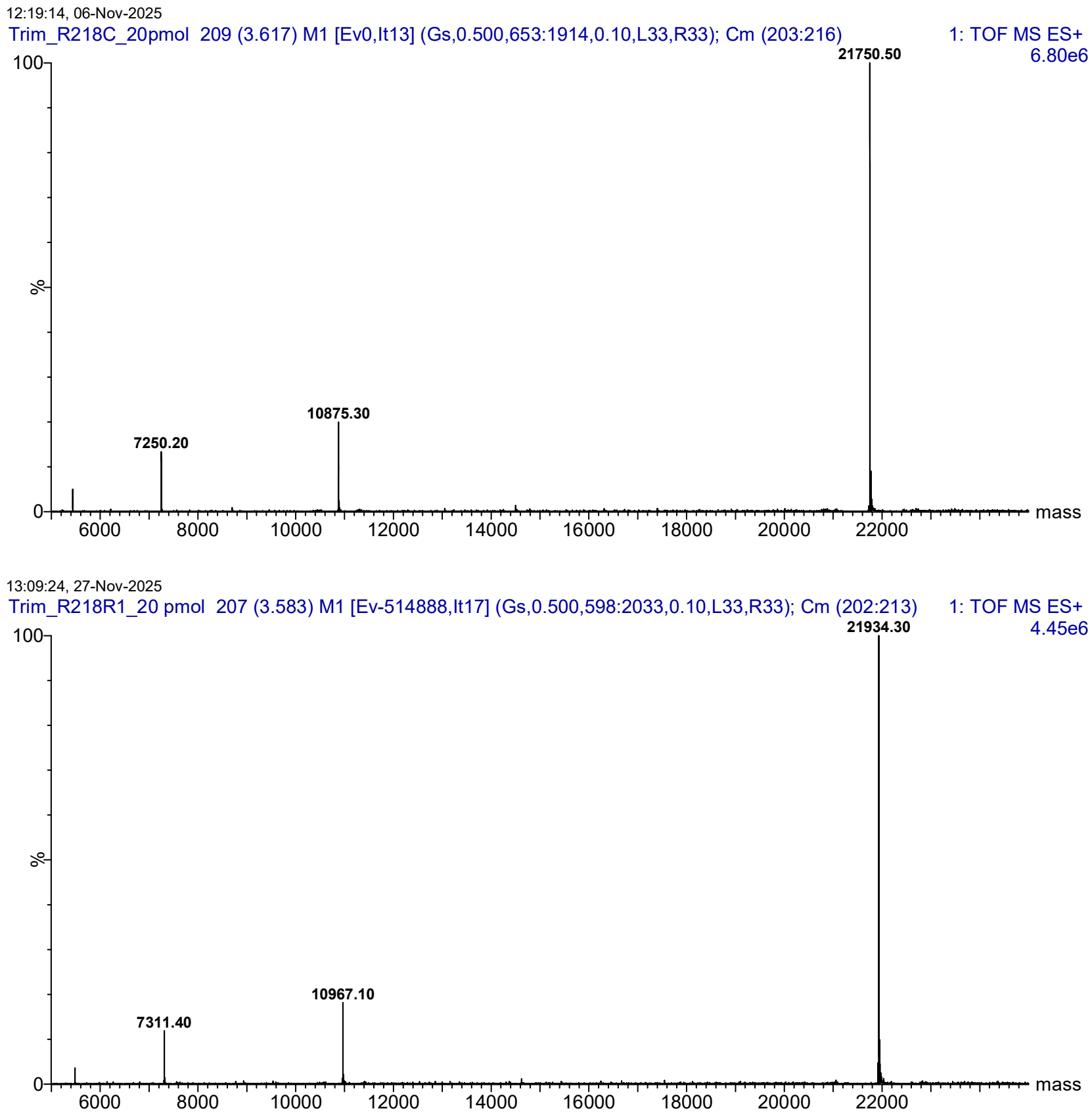

Figure S7: Deconvoluted ESI mass spectrometry data of TRIM25 R218C (top) and R218R1 (bottom).

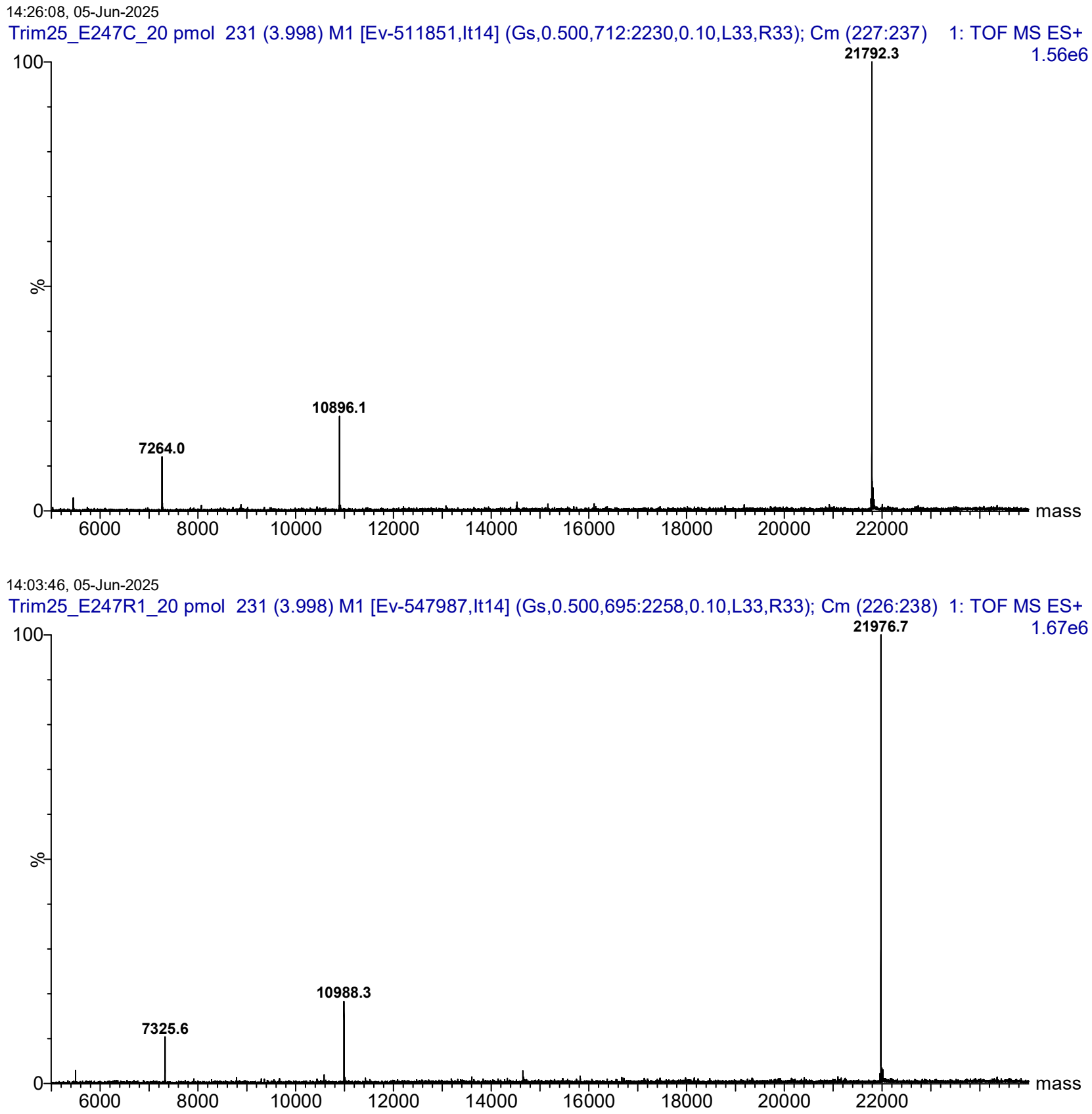

Figure S8: Deconvoluted ESI mass spectrometry data of TRIM25 N214C (top) and N214R1 (bottom).

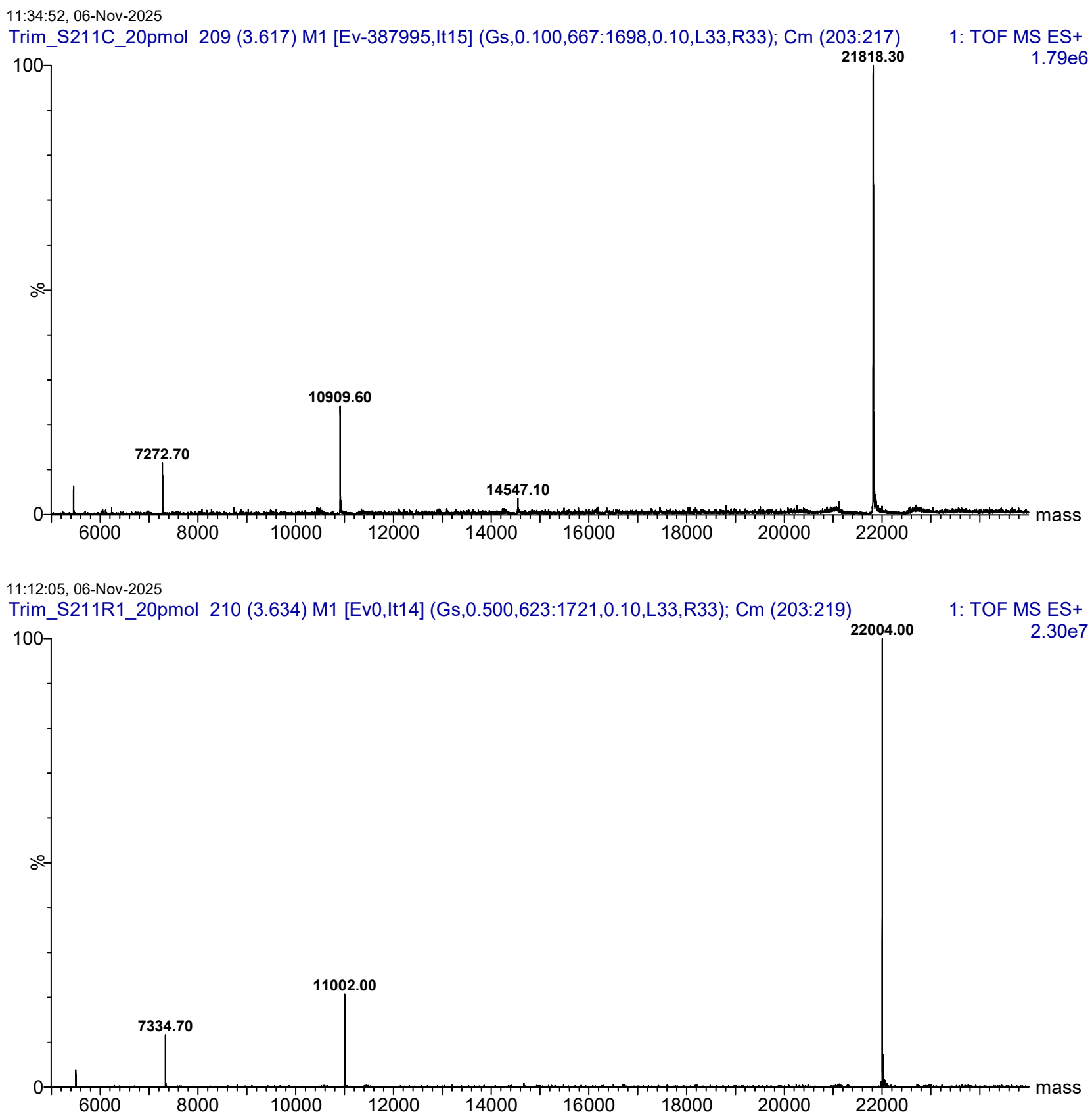

Figure S9: Deconvoluted ESI mass spectrometry data of TRIM25 S211C (top) and S211R1 (bottom).

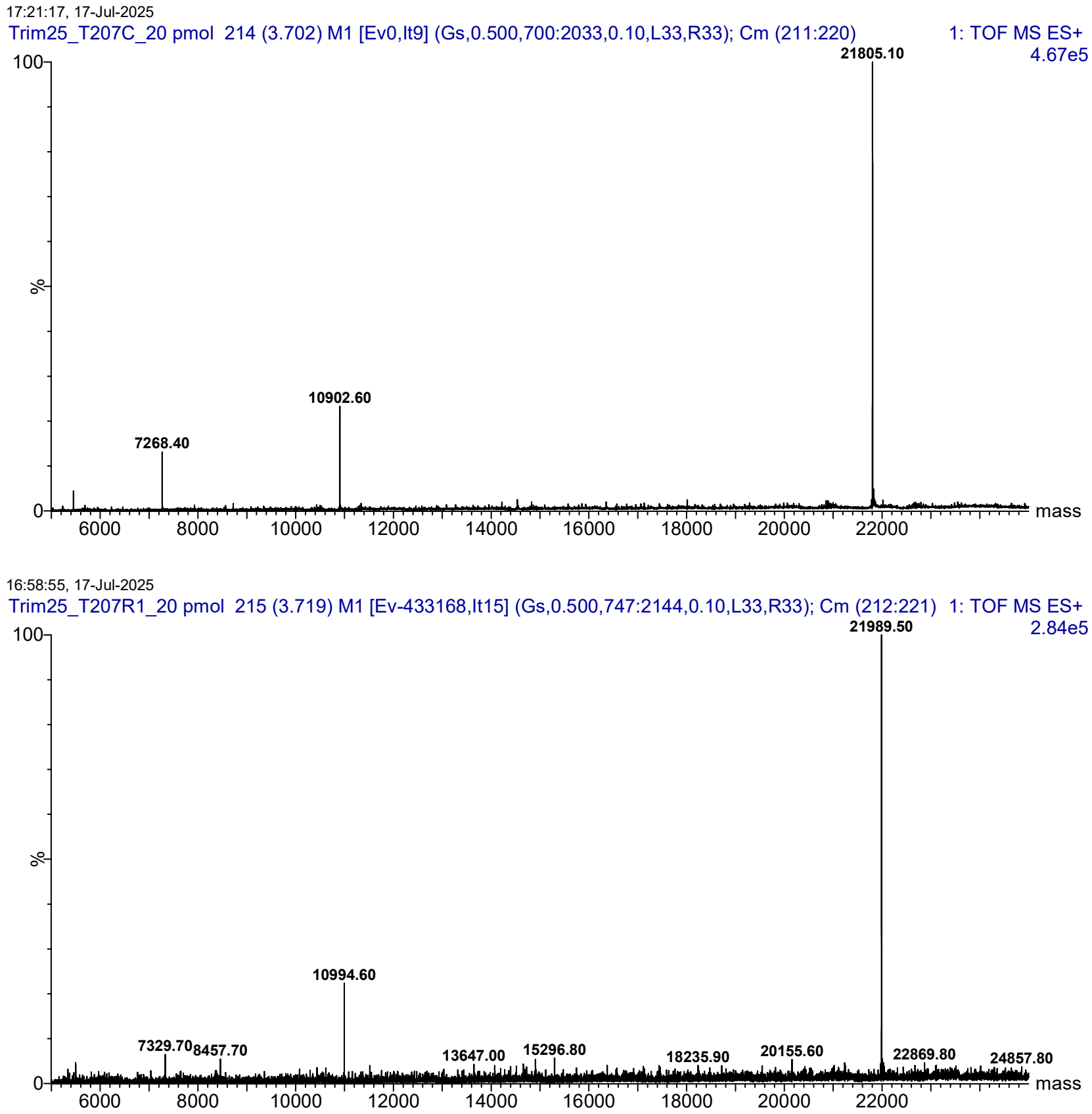

Figure S10: Deconvoluted ESI mass spectrometry data of TRIM25 T207C (top) and T207R1 (bottom).

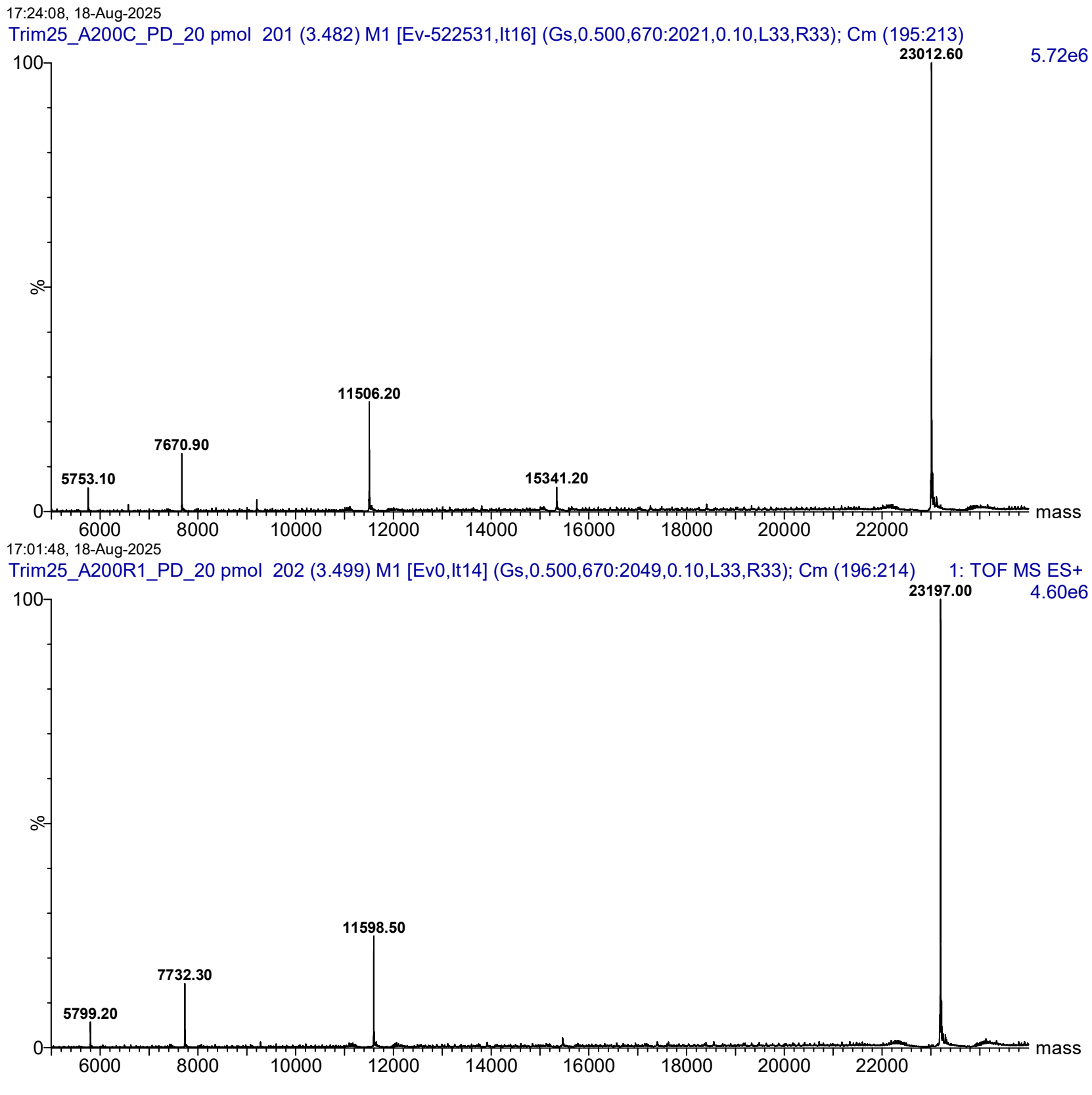

Figure S11: Deconvoluted ESI mass spectrometry data of perdeuterated TRIM25 A200C (top) and A200R1 (bottom).

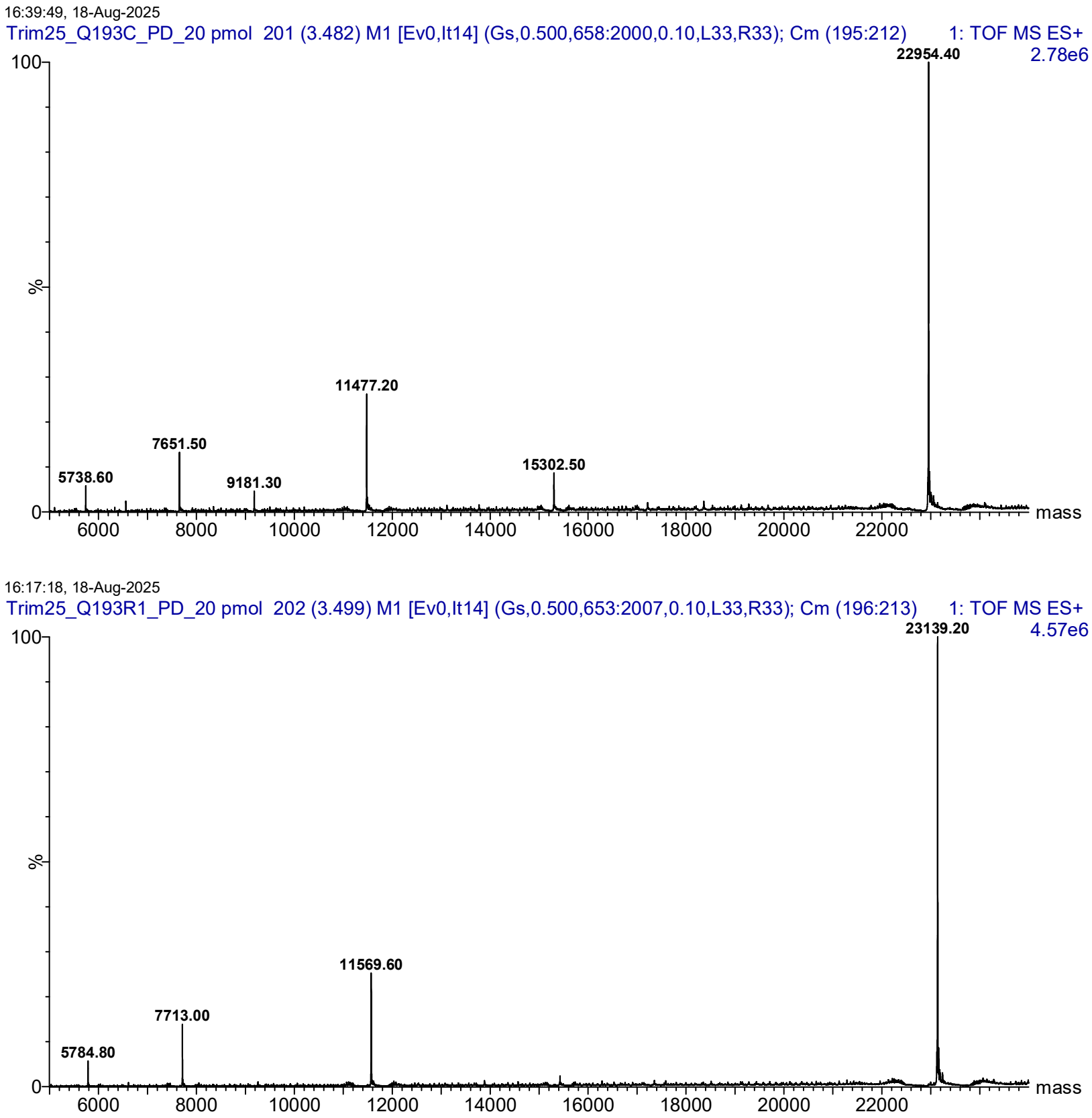

Figure S12: Deconvoluted ESI mass spectrometry data of perdeuterated TRIM25 Q193C (top) and Q193R1 (bottom).

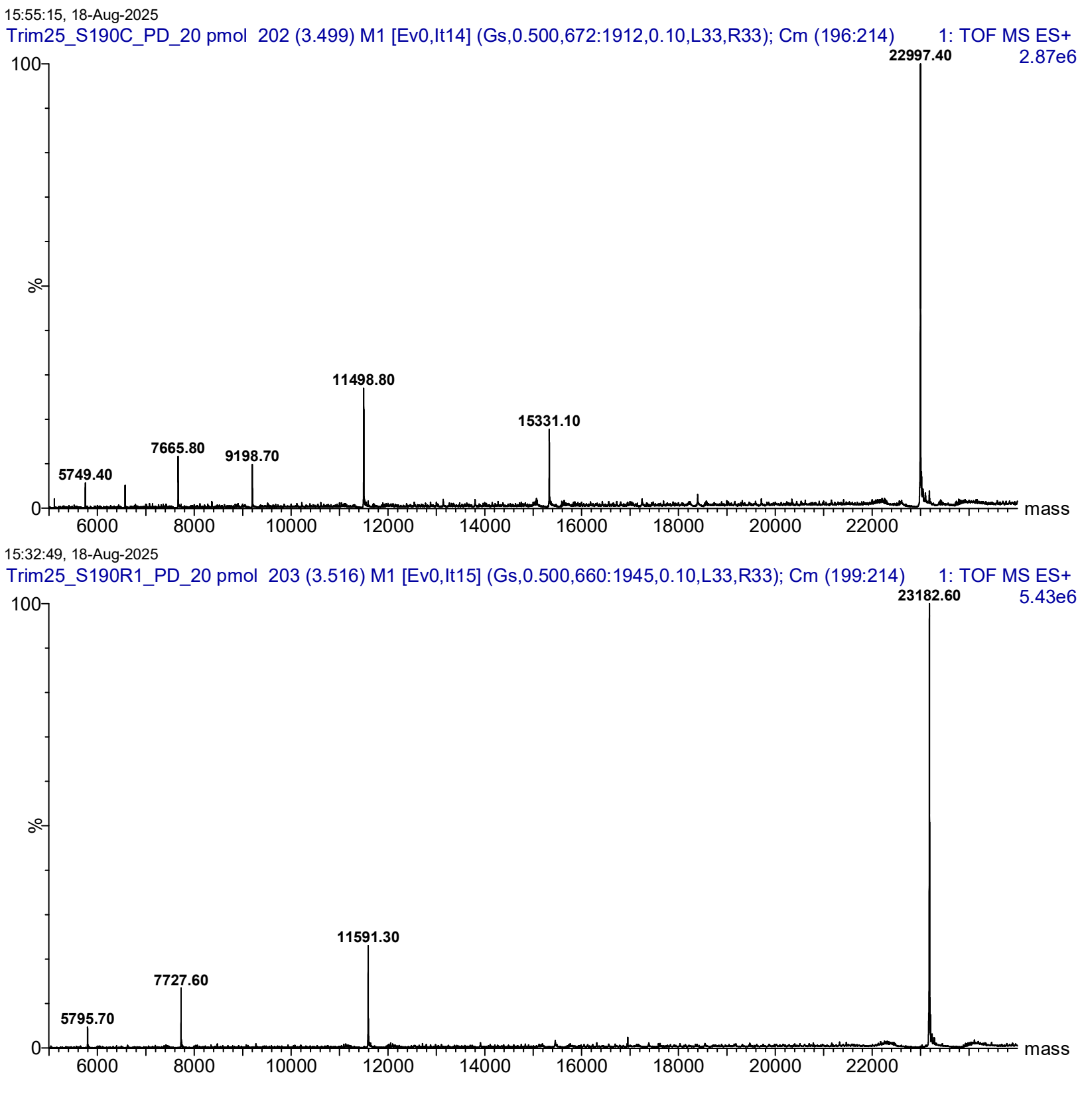

Figure S13: Deconvoluted ESI mass spectrometry data of perdeuterated TRIM25 S190C (top) and S190R1 (bottom).

2.3 CW-EPR spectroscopy

All spin-labeled TRIM25 mutants contain only negligible amounts of free spin-label as visible in Figure S14-15. The measured spin counts result in labeling efficiencies above 80%. Since the amount of available perdeuterated protein was low, no CW spectra were recorded for these samples.

Table S8: Measured spin counts and the respective labeling efficiencies for all non-perdeuterated samples.

Mutant	Spin count / µM	Labeling efficiency / %
D221R1	485	99
R218R1	227	90
N214R1	398	85
S211R1	188	81
T207R1	328	81

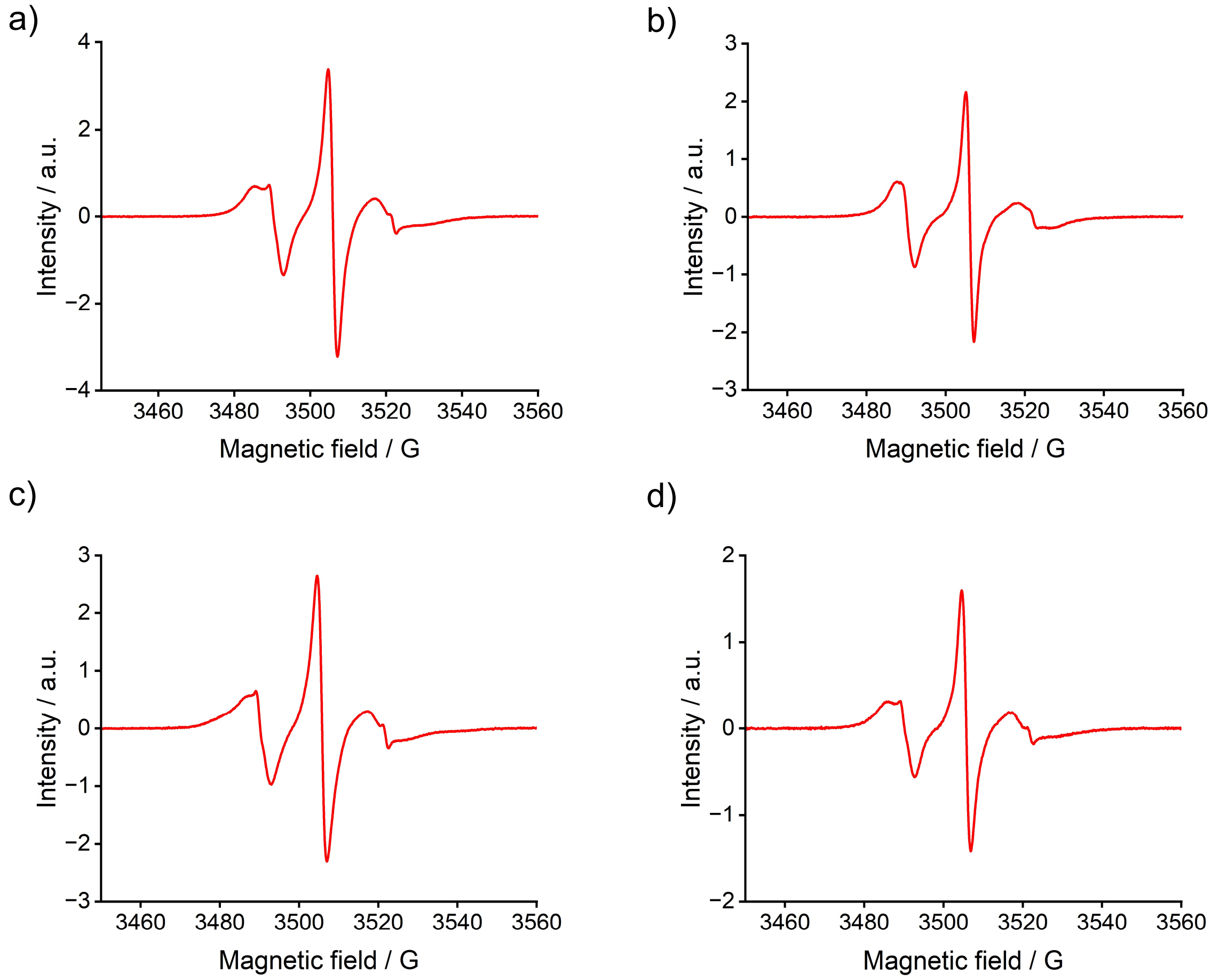

Figure S14: CW-EPR spectra of the MTSL labeled TRIM25 mutants a) D221R1, b) R218R1, c) N214R1 and d) S211R1.

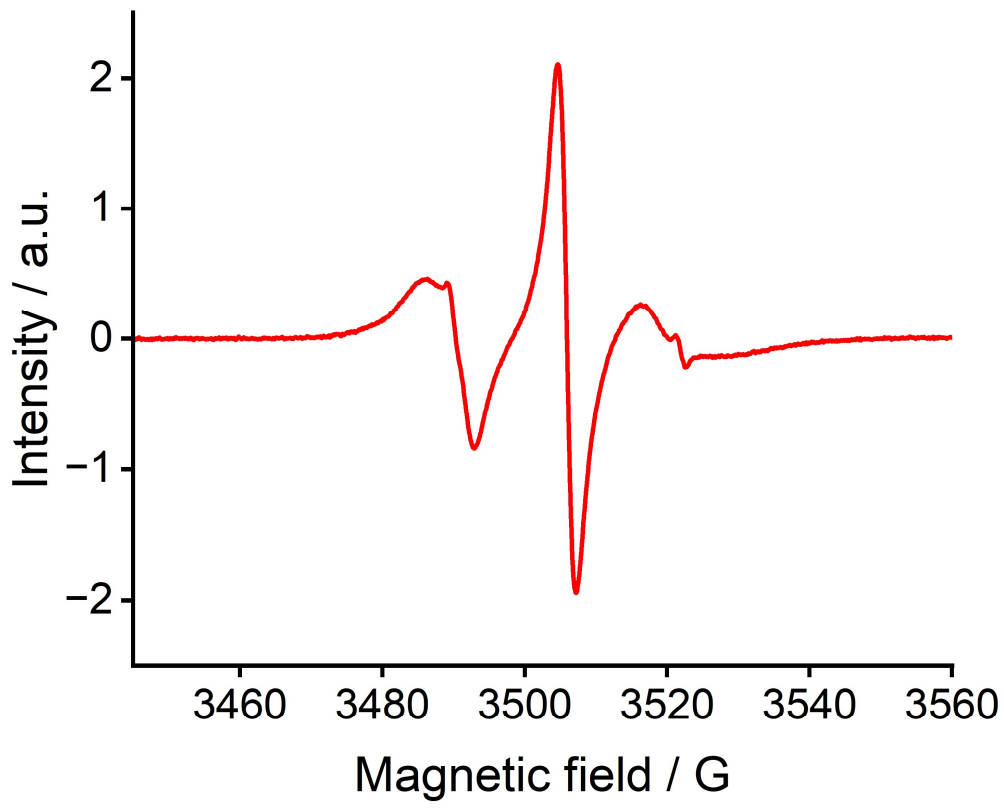

Figure S15: CW-EPR spectrum of the MTSL labeled TRIM25 mutant T207R1.

2.4 Influence of the cryoprotectant on T_m

To assess the influence of the cryoprotectant, a second sample of the N214R1 was measured in presence of 50% d8-glycerol instead of d6-ethylene glycol. The resulting 2p echo decay traces show that the decay is slightly slower in the case of ethylene glycol which has been used for all following samples (Figure S16). The difference between the cryoprotectants is small in case of the N214R1 mutant, some shorter mutants that are not included in this work showed more pronounced effects in favor of ethylene glycol.

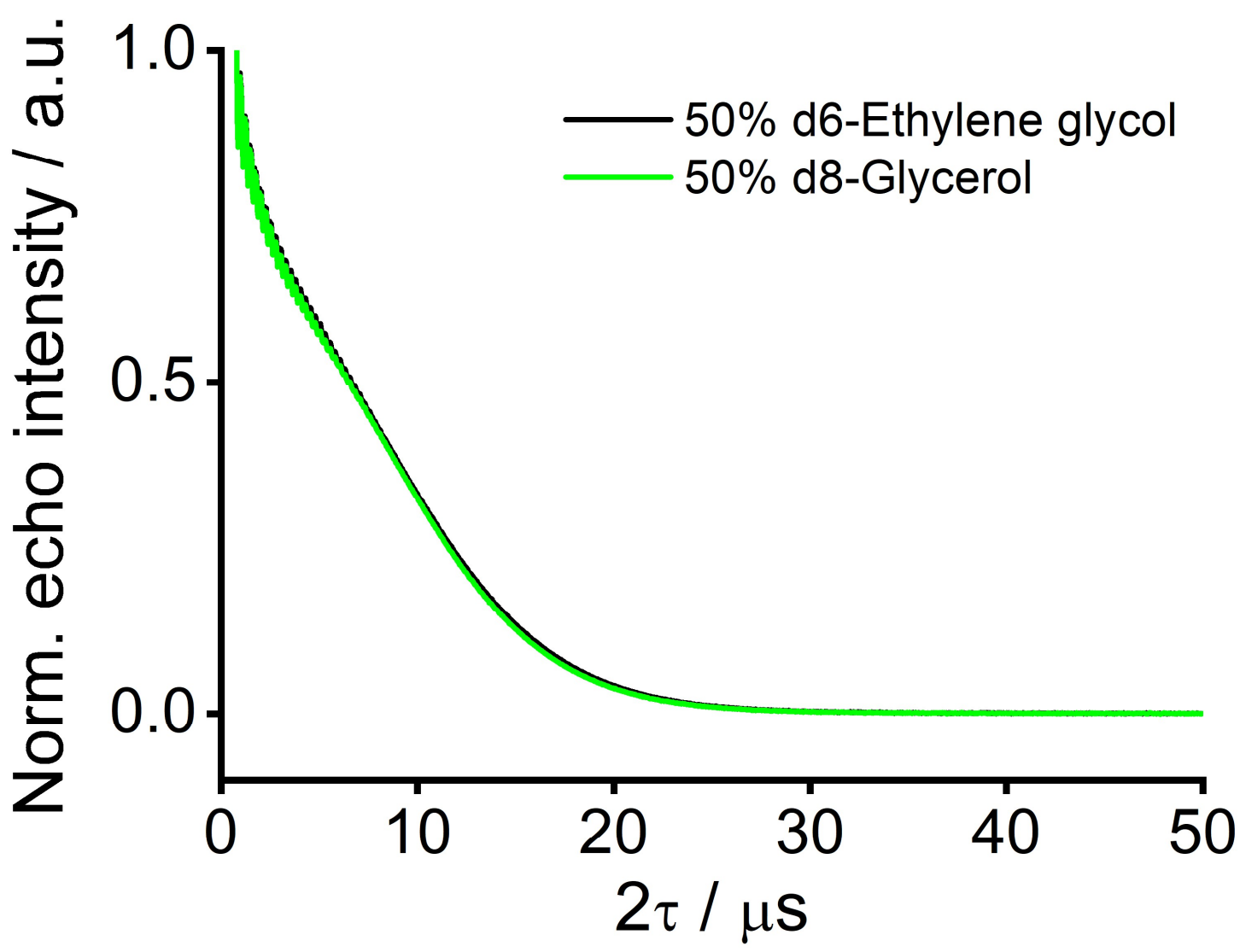

Figure S16: 2p echo decay measurements of TRIM25 mutant N214R1 in 50% d6-ethlyene glycol (black) and d8-glycerol (green) respectively.

2.5 Quantitative description of trace length distance ratio

The necessary trace length to determine the average distance and the width of the resulting distance distribution can be calculated based on the following equations,[4]

$$t_{max} \geq 1.9 \cdot 10^{-5} \cdot r_{max}^{3} \frac{\mu s}{\text{Å}^3}, \tag{3}$$

$$t_{max} \geq 3.8 \cdot 10^{-5} \cdot r_{max}^{3} \frac{\mu s}{\text{Å}^3}, \quad (4)$$

with t_{max} the minimal required trace length for a reliable analysis of a distance r_{max}. Equation (3) describes the necessary trace length to reliably determine the mean and equation (4) to determine mean and width of the distribution. When applying the above equations to the designed TRIM25 mutants, required trace lengths for the respective criteria can be calculated (Figure S17).

a)

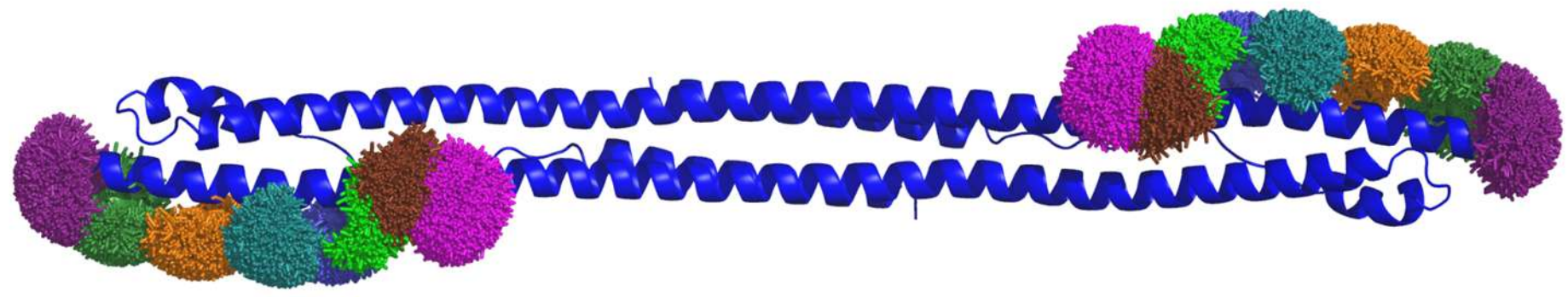

b)

		Trace length / µs	
**Mutant**	<r$_{AV}$> / nm	Mean	Mean and w.
D221R1	8.63	12.2	24.4
R218R1	9.80	17.9	35.8
N214R1	10.47	21.8	43.6
S211R1	11.91	32.1	64.2
T207R1	13.27	44.4	88.8
A200R1	15.33	68.5	136.9
Q193R1	17.27	97.9	195.7
S190R1	18.93	128.9	257.8

c)

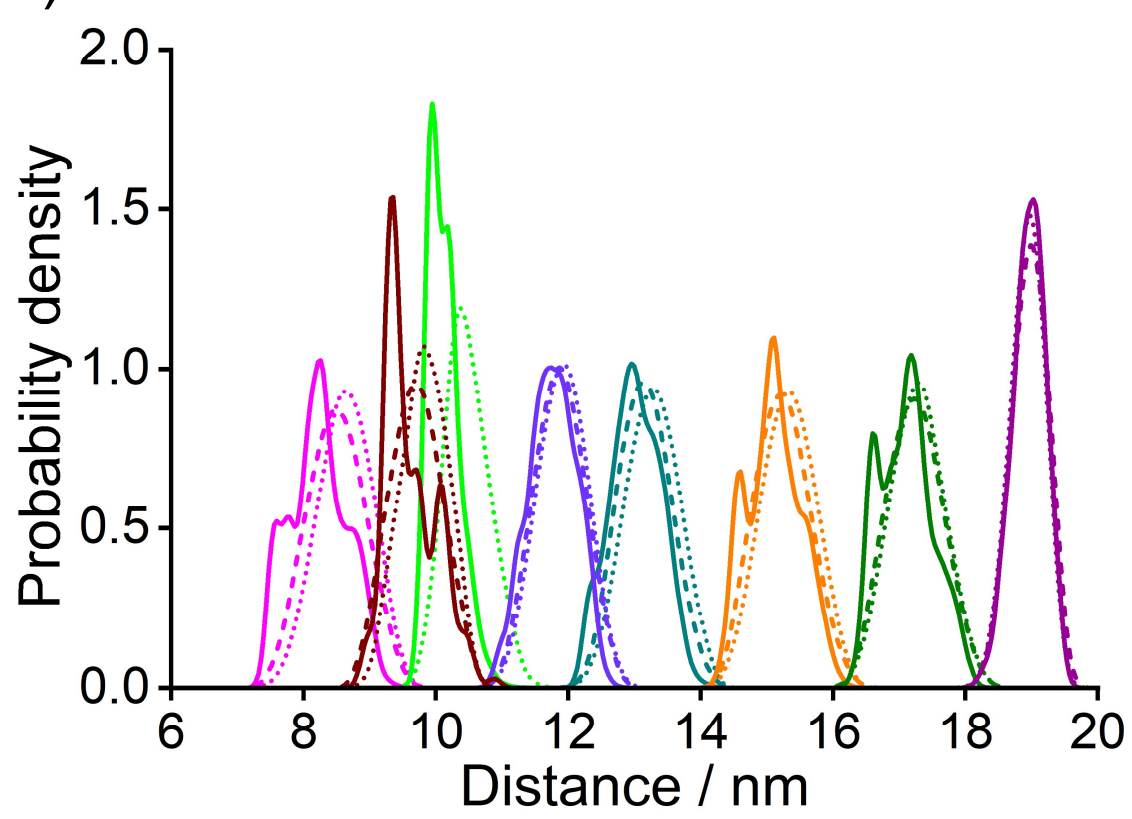

Figure S17: a) Visualization of the crystal structure with the MTSL rotamer clouds calculated *via* the OR approach in ChiLife. b) The expected average distance calculated *via* the AV approach in ChiLife and the respective trace length necessary to accurately evaluate the mean distance or mean and width (w.), based on equations (3+4). c) The predicted distance distributions from ChiLife utilizing the RL (solid), OR (dash) and AV (dot) approaches, in the same color code as in (a).

2.6 DeerLab analysis, comparison between compactness and non-compactness

The DeerLab analysis of the 4p DEER Data (Figure S18) is very stable up to the N214R1 mutant as described in the main text. Using the compactness criterion does not affect the average distances and the respective standard deviations (Table S9). Surprisingly, even for S211R1 a reasonable average distance is extractable. The width of the distribution increases significantly in comparison to N214R1. The resulting distance distribution for T207R1 cannot be interpreted independent of the use of the compactness criterion.

Table S9: Measured averaged distances and standard deviations calculated from the DeerLab fit with compactness (c) and without compactness in comparison to the ChiLife predictions with the RL, OR and AV method.

	N214R1		S211R1		T207R1	
	<r> / nm	σ_r / nm	<r> / nm	σ_r / nm	<r> / nm	σ_r / nm
Exp. (c)	10.32	1.35	11.69	1.45	15.16	2.63
Exp.	10.35	1.15	11.89	1.61	14.56	2.36
RL	10.12	0.24	11.76	0.37	13.02	0.39
OR	10.46	0.33	11.87	0.38	13.16	0.40
AV	10.47	0.34	11.91	0.38	13.27	0.41

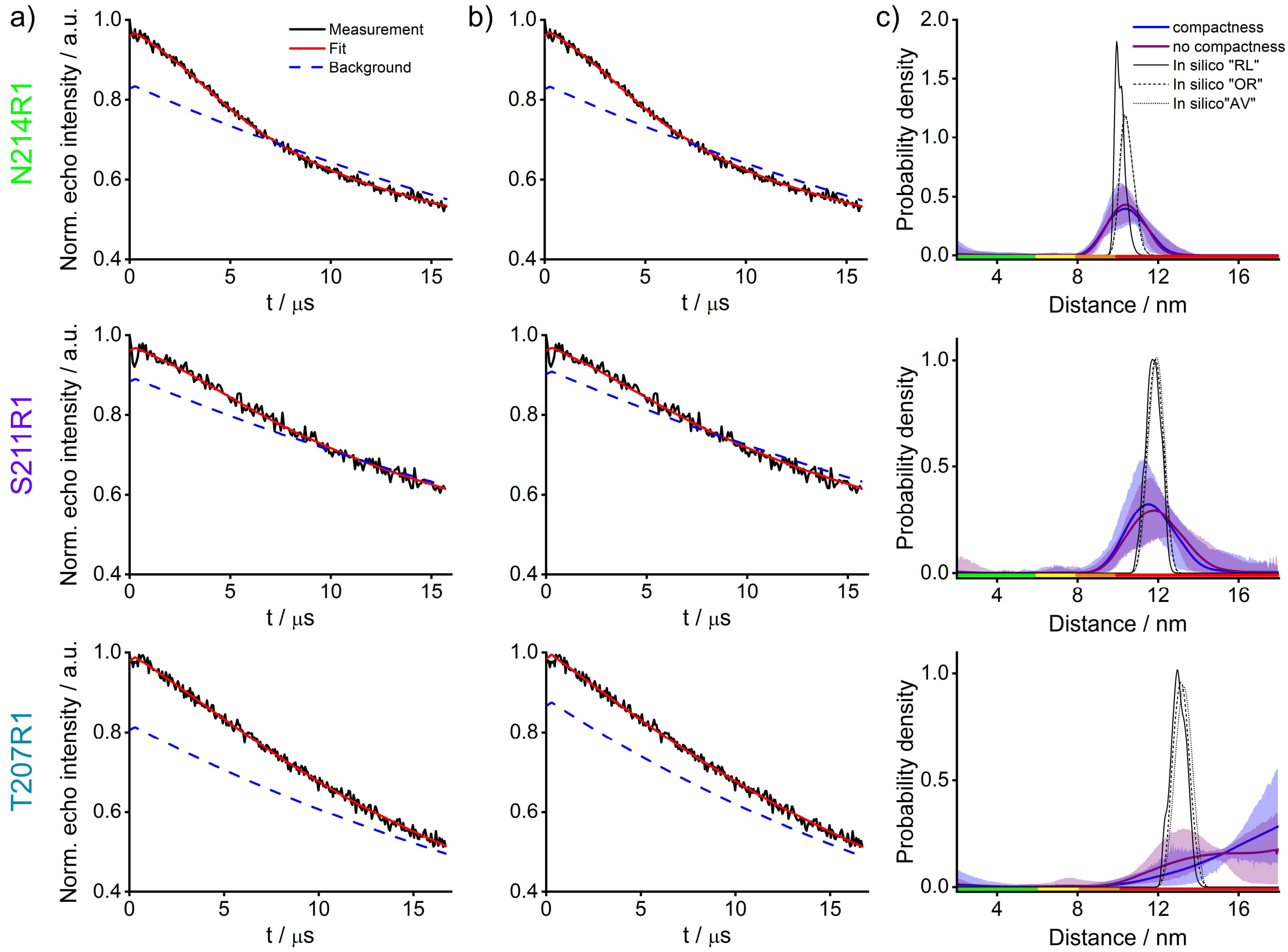

Figure S18: 4 pulse DEER measurements at 50 K of the TRIM25 mutants N214R1 (top), S211R1 (middle) and T207R1 (bottom) with increasing average distance from top to bottom. a) Time traces, analyzed in DeerLab with single pathway analysis. Depicted is the measurement (black), the resulting fit (red) and the background contribution (dash, blue). b) Time traces, analyzed in DeerLab with single pathway analysis and compactness criterion, in the same color code as in (a). c) Resulting distance distributions with (blue) and without (purple) compactness criterion as well as the predicted distance distributions from ChiLife utilizing the RL (solid, black), OR (dash, black) and AV (dot, black) approaches. The reliability of the distance distributions is depicted by the color bars from green to red.

2.7 5p DEER measurements

By utilizing 5p DEER measurements the accessible trace length could indeed be prolonged by a factor of about 1.4, e.g. in case of the N214R1 (Figure S20, top) from 16 µs to about 22 µs. This can be visualized by looking at the respective refocused echo decay of the 5p measurement in comparison to the 4p measurement (Figure S19, top). Whereas for the N214R1 mutant in deuterated solution a significant gain in echo intensity is observed, effects on perdeuterated mutants such as S190R1 (Figure S19, bottom) are negligible. This highlights that in case of very high deuteration degrees such as with perdeuterated samples no significant gain in accessible time window can be achieved by utilizing the 5p DEER sequence.

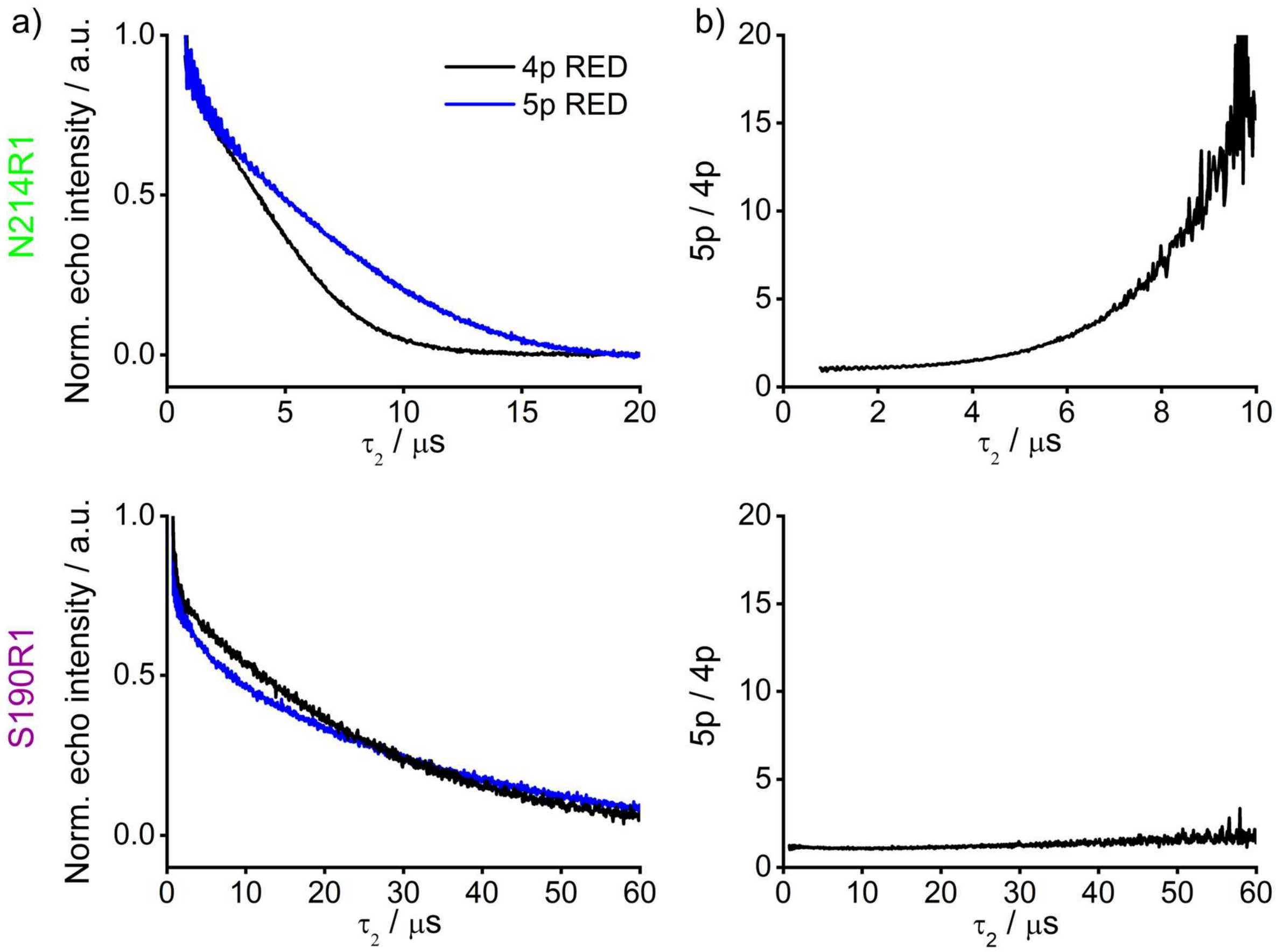

Figure S19: Refocused echo decay (RED) experiments of TRIM25 mutants N214R1 (top) and S190R1 (bottom). a) Comparison between a 4p and 5p RED and b) Relative gain in echo intensity in dependence of τ.

In case of the non-deuterated mutants, specifically the N214R1, the resulting distance distributions fit nicely to the predicted distances independent of whether the compactness criterion was applied. Since 5p DEER traces show a characteristic feature from one of multiple possible dipolar pathways, a multi component pathway analysis needs to be conducted. Usually, if the excitation overlap between the pump and probe pulses is low at least two pathways (pathway 1 and 5) need to be fitted to describe the 5p experiment, this feature was implemented in DeerLab[9] and was utilized for the analysis of the 5p data. Interestingly, pathway two is the most dominant in the analysis of all measured traces which is unexpected

since pathway five gives rise to the artifact in the middle of the 5 pulse time trace.[9] This can only be attributed to the comparably short trace length in relation to the dipolar oscillation frequency and the thereby high uncertainty in the analyses. Especially for the two longer distance mutants S211R1 and T207R1 (Figure S20, middle and bottom) use of the compactness criterion tends to shift the distance distributions to shorter distances. This highlights that the data is probably over-interpreted and should be treated carefully. Nevertheless, since T207R1 featured a more beneficial relaxation behavior and the 5p trace could be recorded over a longer time window the resulting distance distribution, especially when the compactness criterion is not applied, still matches the expected average distance nicely.

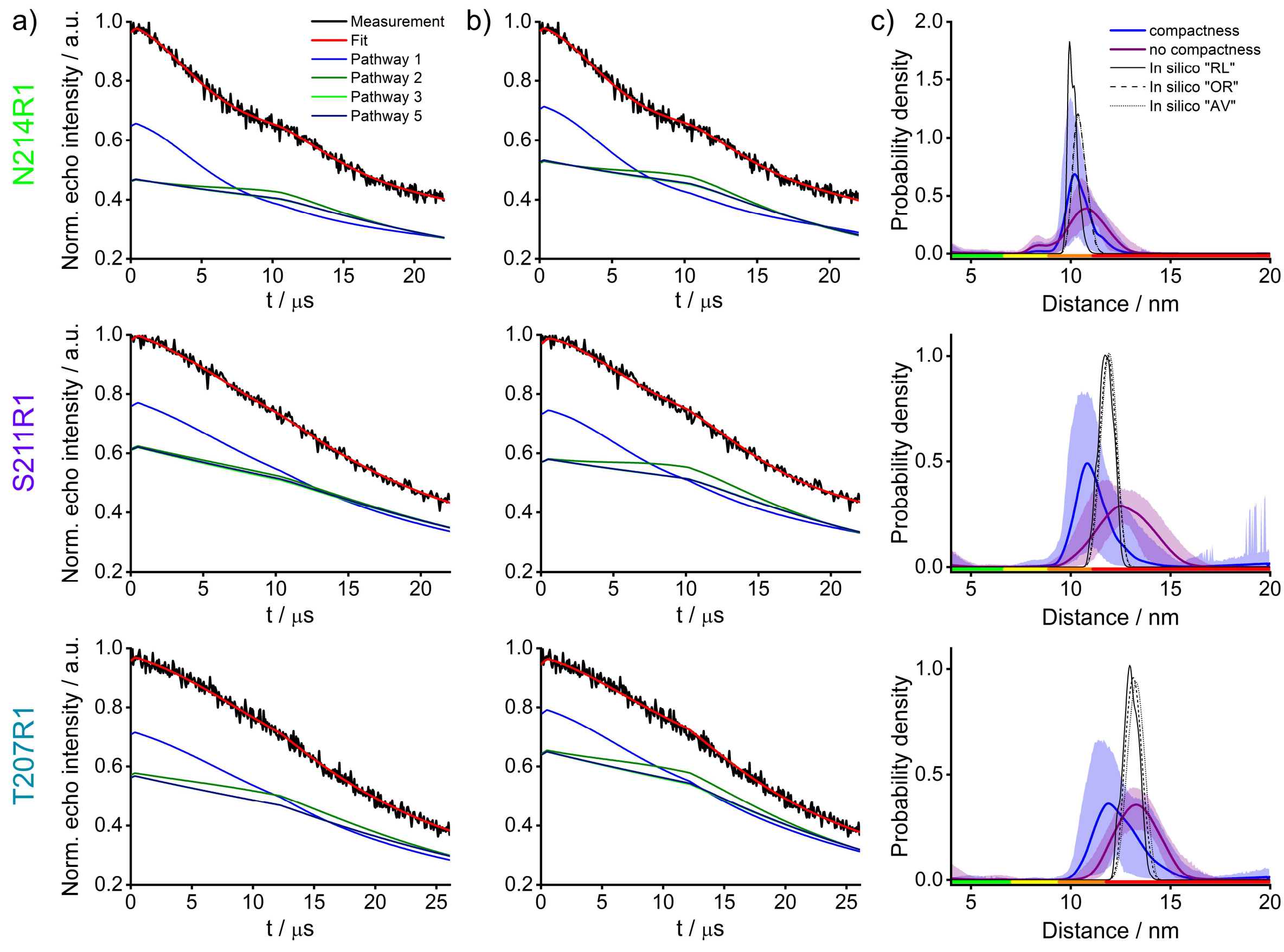

Figure S20: 5 pulse DEER measurements at 50 K of the TRIM25 mutants N214R1 (top), S211R1 (middle) and T207R1 (bottom) with increasing average distance from top to bottom. a) Time traces, analyzed in DeerLab with multi pathway analyses. Depicted is the measurement (black), the resulting fit (red) and the pathway contributions 1 (blue), 2 (olive), 3 (green), 5 (dark blue). b) Time traces, analyzed in DeerLab with multi pathway analyses and compactness criterion, in the same color code as in (a). c) Resulting distance distributions with (blue) and without (purple) compactness criterion as well as the predicted distance distributions from ChiLife utilizing the RL (solid, black), OR (dash, black) and AV (dot, black) approaches. The reliability of the distance distributions is depicted by the color bars from green to red.

2.8 Relaxation time measurements and optimal SNR for perdeuterated samples

To assess the influence of protein deuteration on the expected sensitivity optimum for the PELDOR experiment, a set of inversion recovery and 2p echo decay experiments has been conducted (Figure S21) as described in section 1.8.2. This set of measurements was conducted on a 10 µM sample of S190R1. The expected signal to noise ratio (SNR) can be calculated as previously described[15] with the following equation,

$$SNR_{PELDOR} \sim \frac{1}{T} \cdot \exp\left(\frac{-2t_{max}}{T_m}\right) \cdot \frac{1}{\sqrt{T_1}} \tag{5}$$

with T the Temperature, t_{max} the dipolar evolution time window, T_2 and T_1 the previously described relaxation times. T_2 is not directly extractable from the 2p echo decay experiment and the phase memory time T_m will be used instead. If a stretched exponential is assumed to fit the phase memory time, the equation can be slightly modified,

$$SNR_{PELDOR} \sim \frac{1}{T} \cdot \exp\left(-\left(\frac{2t_{max}}{T_m}\right)^b\right) \cdot \frac{1}{\sqrt{T_1}} \tag{6}$$

with b the respective stretching exponent. The fitted T_1 and T_m values are presented in Table S10 with the calculated SNR values (Table S11) for a t_{max} of 10 µs and 100 µs.

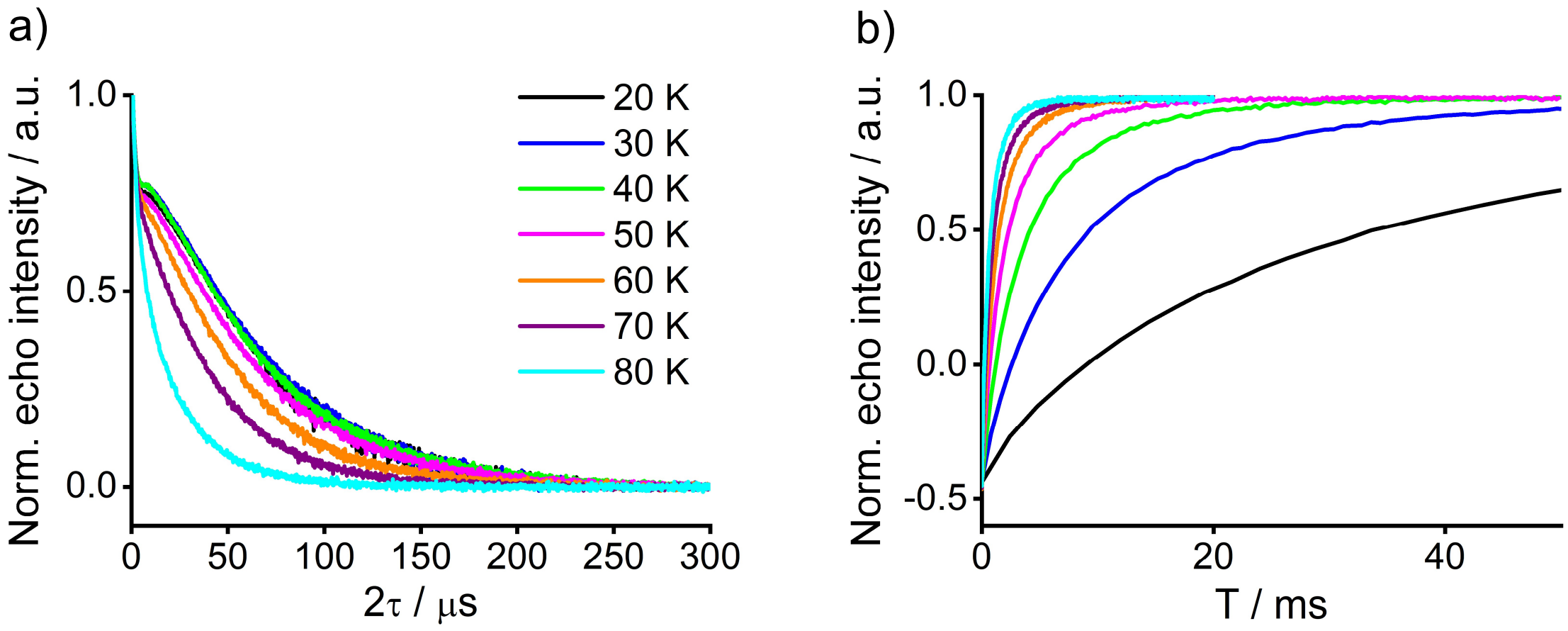

Figure S21: Temperature dependent a) 2p echo decay and b) inversion recovery measurements.

Table S10: T_1 and T_m values fitted with mono (m) or stretched (s) exponential functions.

T / K	T_1 / ms	T_m (m) / us	T_m (s) / us	b (s)
20	39.2	66.4	74.0	1.29
30	10.5	67.4	75.0	1.29
40	4.58	65.6	71.6	1.22
50	2.56	61.2	66.3	1.18
60	1.57	49.8	53.4	1.13
70	1.15	37.5	36.5	0.96
80	0.85	19.2	12.0	0.67

Table S11: Calculated SNR values based on T_m values fitted with mono (m) or stretched (s) exponential functions with a t_{max} of 10 and 100 µs respectively.

T / K	$SNR_{norm.}$ (10 µs, m)	$SNR_{norm.}$ (100 µs, m)	$SNR_{norm.}$ (100 µs, s)
20	0.65	0.71	0.61
30	0.85	0.95	0.85
40	0.96	1.00	1.00
50	1.00	0.86	0.89
60	0.99	0.43	0.43
70	0.87	0.12	0.22
80	0.53	0.00(072)	0.06

For better visualization the respective SNR and relaxation times were plotted against the temperature (Figure S22). Assuming a short distance would be of interest and the time window is short, 50 K is still the sensitivity optimum for PELDOR experiments as described in the literature.[15] Since the samples with deuterated protein feature long distances the time window will be long and at least 100 µs. In this case the second term of equation (3) becomes dominant leading to a shift of the sensitivity optimum to 40 K. The gain in comparison to measuring at 50 K lies between 11-14% (depending on the fitting of T_m) which is not negligible. Therefore, all measurements of perdeuterated protein were conducted at 40 K.

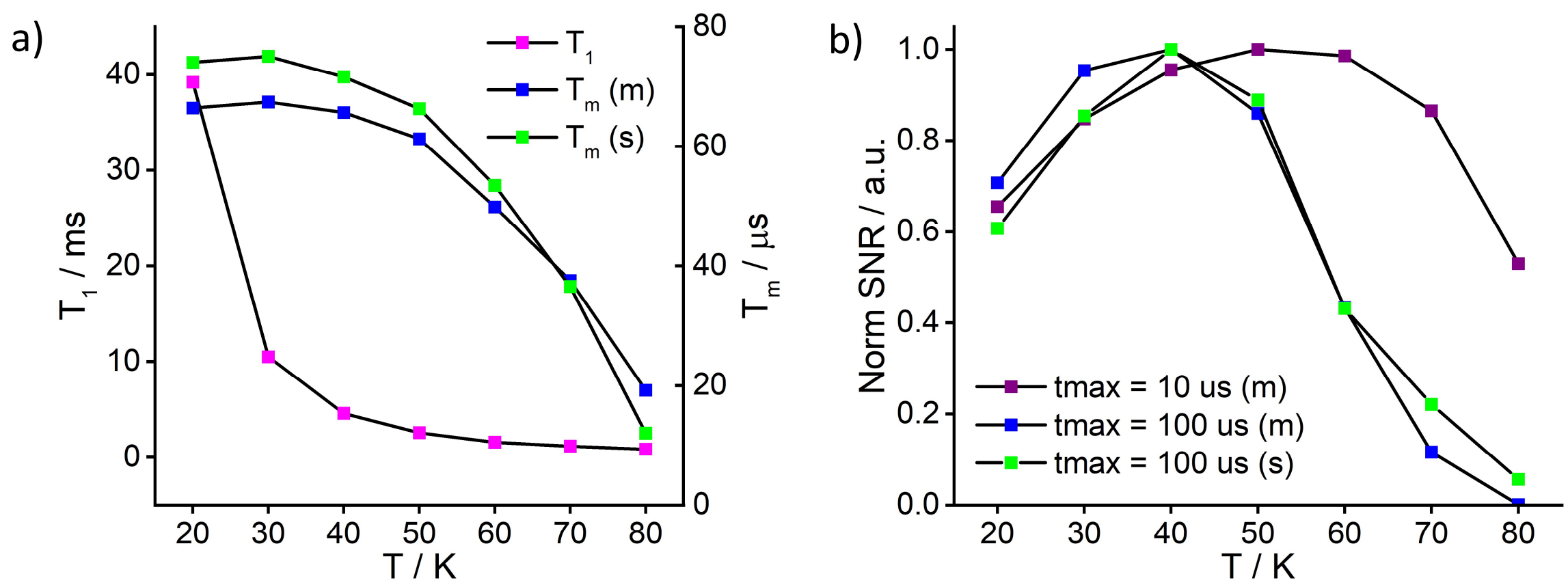

Figure S22: a) Fitted T_1 (magenta), T_m mono (m) exponential (blue) and T_m stretched (s) exponential (green) values with respect to the temperature and b) the calculated SNR values with a t_{max} of 10 µs (purple), 100 µs (m) (blue) and 100 µs (s) (green).

2.9 Concentration considerations

The sample concentration becomes relevant for measuring long range distance constraints due to instantaneous diffusion, decreasing the echo intensity of the refocused echo in 4p DEER. The optimal concentration can be calculated as previously described,[15]

$$c_{opt} = \frac{9\sqrt{3}}{2\pi} \cdot \frac{\hbar}{t_{max}\, f_{A,\pi}\, N_A\, g_A\, g_B\, \mu_0\, \mu_B{}^2} \tag{7}$$

with t_{max} the maximum dipolar evolution time to resolve two oscillations, $f_{A,\pi}$ which can be approximated to 0.25 in case of 32 ns observer π pulses, $g_A\ g_B$ the g-values of the observed and the pumped spins, respectively, and c_{opt} the optimal spin concentration. Based on this

formula optimal concentrations can be calculated for the accessible t_{max} and following from that also for the expected distance (Table S12).

Table S12: Calculated trace length and optimal concentrations in the distance range between 10-20 nm. The distances corresponding to mutants are color coded respectively.

<r> / nm	t_{max} / µs	c_{opt} / µM
10.0	38.3	104
13.3	90.1	44.4
13.7(8)	100	40.0
15.3	137	29.1
17.3	198	20.2
18.9	259	15.5
20.0	306	13.0

To confirm this experimentally a set of differently concentrated samples of mutant S190R1 were prepared with 30 µM, 10 µM, 3 µM, 1 µM and RED measurements were conducted (Figure S23).

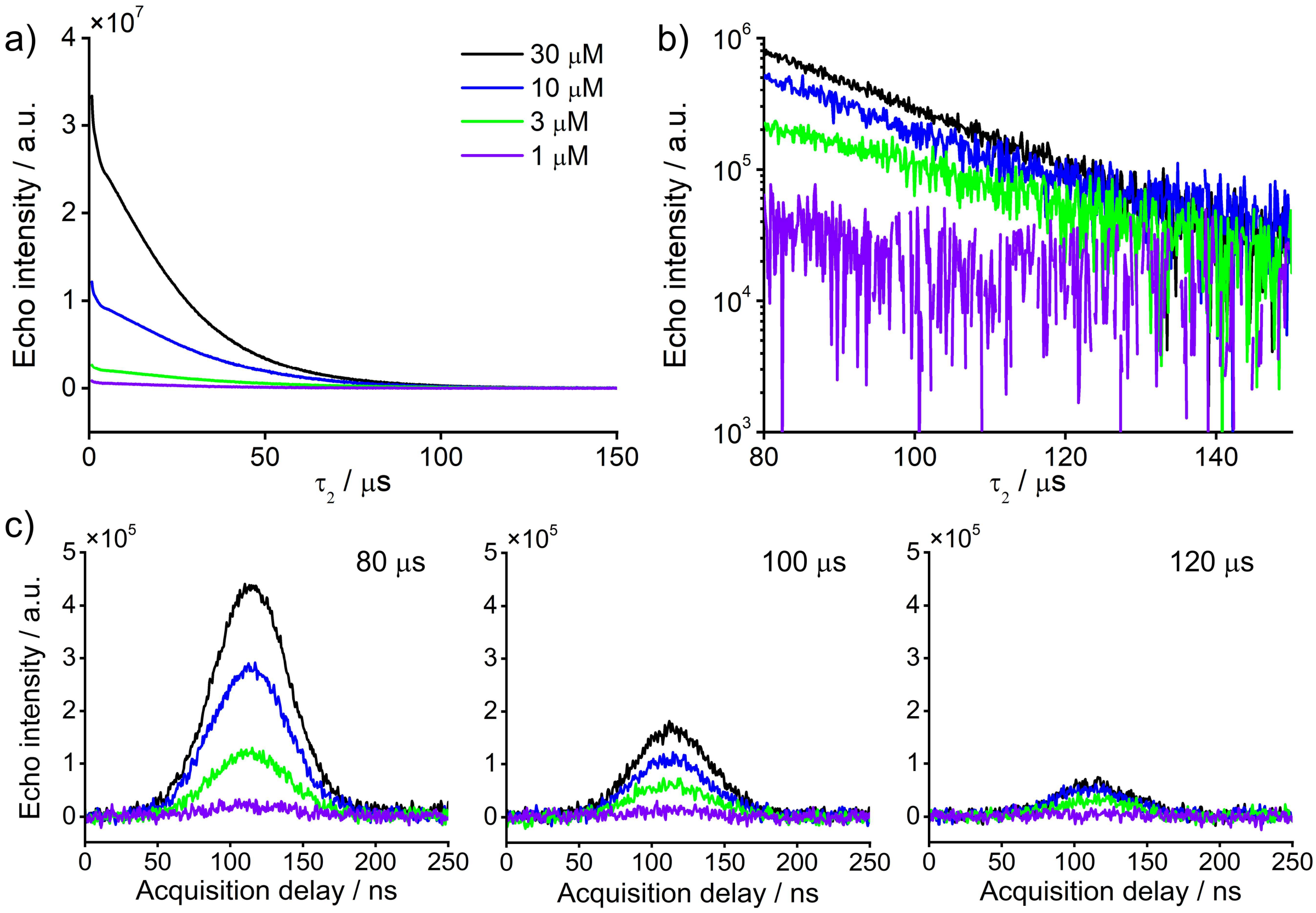

Figure S23: Concentration dependent measurements at 40 K with the perdeuterated TRIM25 mutant S190R1 with 30 µM (black), 10 µM (blue), 3 µM (green) and 1 µM (violet), respectively. a) RED, b) a logarithmic zoom into the data in (a) and c) transient echo intensities of 2000 averages at a τ_2 of 80, 100 and 120 µs.

The data shows that at about 120 µs the signal of the 10 µM sample is comparable to the 30 µM sample confirming the calculations. Since the accessible t_{max} for the deuterated samples is about 100 µs or slightly above the concentration of 40 µM spins should be optimal and was used.

2.10 3p/4p DEER comparison

The 3p DEER sequence should provide a sensitivity improvement in comparison to the 4-pulse sequence due to instantaneous diffusion related losses caused by the additional pulse. This is usually not considered favorable over 4p DEER because the latter allows accurate determination of the zero time which is important when looking at short distances (in this case <10 nm). For samples with deuterated protein, small changes in the zero time should not affect the resulting distance distributions since the period of the oscillation due to dipolar coupling is long (Figure S24).

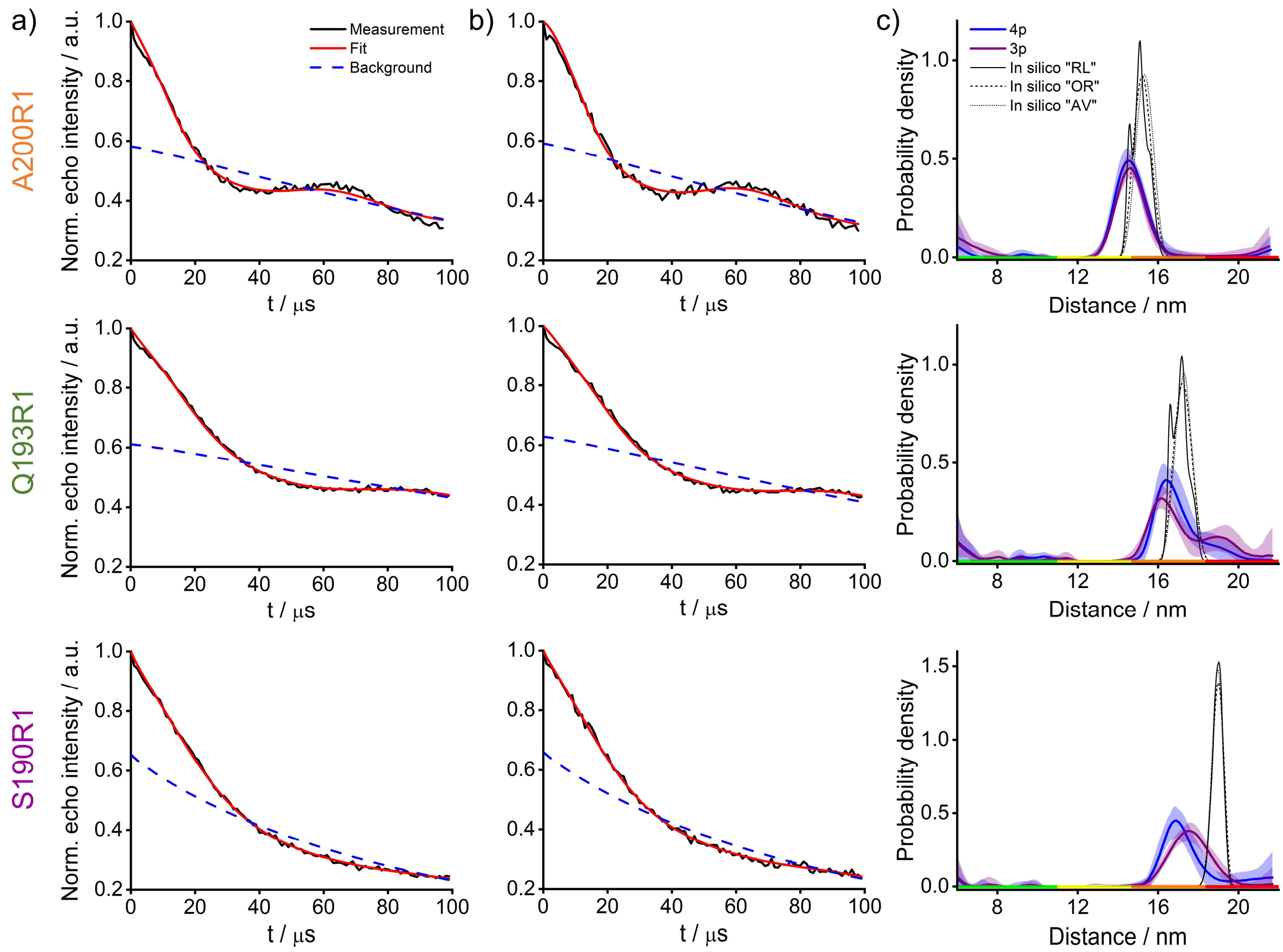

Figure S24: PELDOR experiments at 40 K of the TRIM25 A200R1 (top), Q193R1 (middle), S190R1 (bottom) and with increasing average distance from top to bottom. a) 3p DEER time traces, analyzed with the CDA. Depicted is the measurement (black), the resulting fit (red) and the background contribution (dash, blue). b) 4p DEER time traces, analyzed with the CDA, in the same color code as in (a). c) Resulting distance distributions of 4p DEER (blue) and 3p DEER (purple) as well as the predicted distance distributions from ChiLife utilizing the RL (solid, black), OR (dash, black) and AV (dot, black) approaches. The reliability of the distance distributions is depicted by the color bars from green to red.

The resulting traces show comparable distance distribution in all cases. Noteworthy is the higher SNR (Table S13) for the 3p DEER which enables the acquisition of even longer time traces, resulting in the possible application of PDS to even longer distances. The SNR was adapted from the CDA output.

Table S13: Comparison of the mean distances <r> the respective standard deviation σ_r, the SNR and the SNR normalized by the square root of the acquisition time (SNR_t). All values were calculated from the respective CDA output.

Mutant	Sequence	<r> / nm	σ_r / nm	SNR	Acq. Time / h	SNR_t / $h^{-1/2}$
A200R1	4p	14.58	0.46	31.8	15.9	7.98
	3p	14.56	0.22	43.6	18.2	10.3
Q193R1	4p	16.92	0.93	54.3	21.2	11.8
	3p	17.09	1.37	84.5	22.0	18.0
S190R1	4p	17.33	0.83	36.5	19.7	8.22
	3p	17.61	0.75	53.6	19.1	12.3

The gain of the 3p is especially high for the S190R1 mutant, which can be explained by the slightly higher concentration of this mutant which leads to more pronounced instantaneous diffusion effects, thereby making the 3-pulse sequence particularly useful in this case. The mean distances and standard deviations between the sequences is very consistent underlining that the exact estimation of the zero time is not particularly important in the distance regime of the measured samples.

2.11 Maximal accessible trace length 3p/4p DEER

All previously presented samples with deuterated protein have been measured for less than 24 h, leaving some room for longer averaging times and increasing the time trace length. As visible in Table S13 the Q193R1 mutant features the best SNR_t and was therefore chosen to assess the maximal trace length with 3p/4p DEER measurements. A trace with a length of 150 µs could be recorded (Figure S25) with an SNR≈9 (Table S14) depending on the utilized modulation depth calculation. The 3p DEER trace was longer with 155 µs and an SNR≈8. Both traces are slightly below the recommended SNR value of 10,[4] but this could easily be increased by shortening the time window slightly by a few µs in both cases. In theory about 20 nm distances should be extractable from a trace of this length.

To assess the signal to noise ratio (SNR) in this case, the modulation depth λ of PELDOR experiments was analyzed with the python package DeerLab[7] or *via* Tikhonov analysis in DeerAnalysis2022. In case of the DeerLab calculation, raw traces were phase corrected and a dipolar model with a 3D homogeneous background was fitted to the data. To generate reliable error estimates for the modulation depth values, bootstrapping with 100 cycles was performed. The noise was calculated as root mean square deviation (RMSD), estimated from the second and third quartile of the imaginary part of the phase-corrected trace. The SNR was then weighted by the square root of the acquisition time *t*,

$$SNR_t = \frac{\lambda}{RMSD} \cdot \frac{1}{\sqrt{t}}. \quad (8)$$

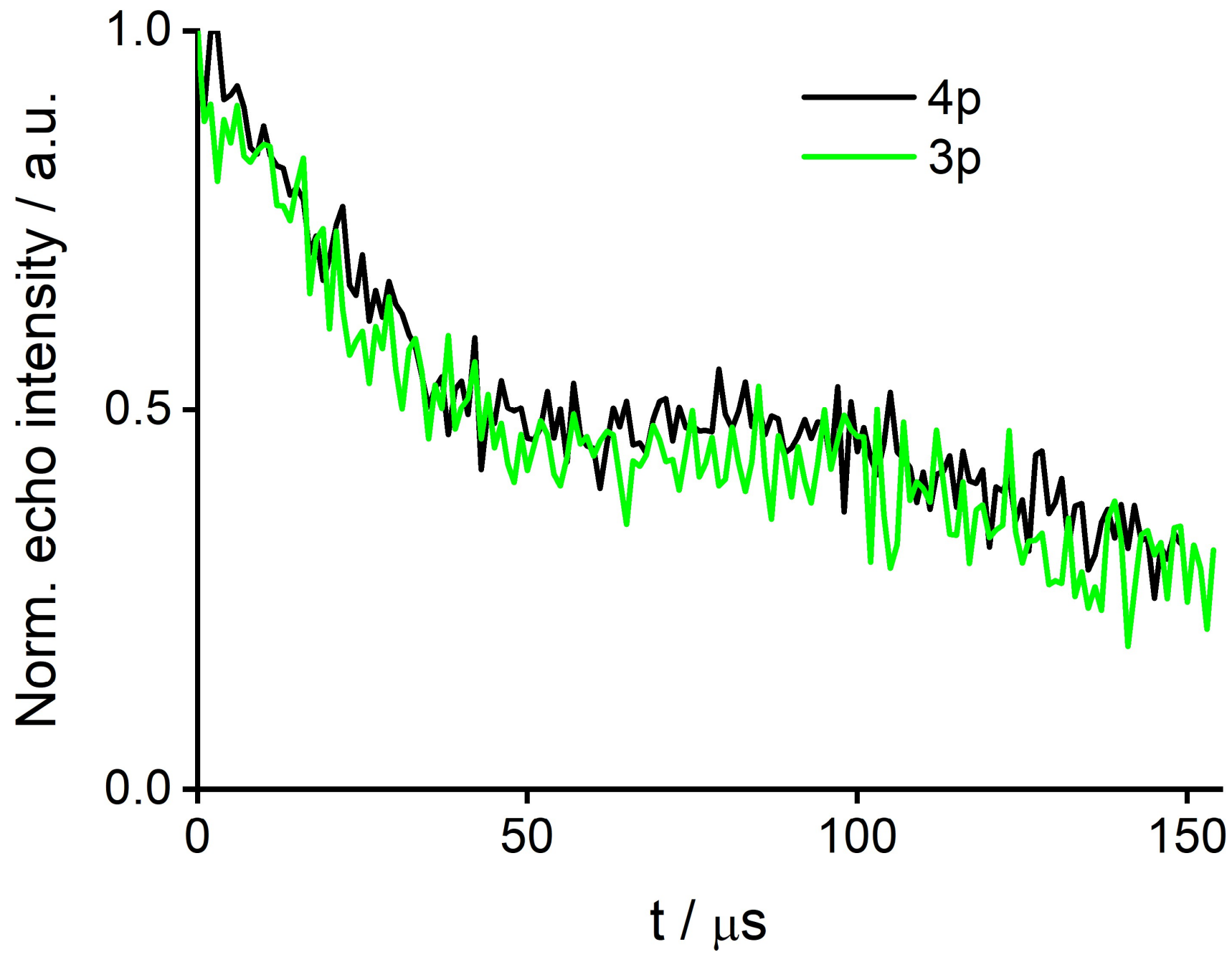

Figure S25: PELDOR experiments at 40 K of the Q193R1 mutant, depicting the 150 µs 4p measurement (black) and 155 µs 3p measurement (green).

Table S14: Calculated SNR for the 150/155 µs measurement in Figure S25 and the theoretical distance $\langle r_{max} \rangle$ that can be reliably extracted from the respective trace length. SNR calculations are based on modulation depths estimated from a DeerLab (DL) and Tihkonov (T) fit.

Mutant	Sequence	λ(DL)	λ(T)	SNR_{imag}(DL)	SNR_{imag}(T)	t_{max} / µs	$\langle r_{max} \rangle$ / nm
Q193R1	4p	0.314	0.324	8.92	9.20	150	19.91
	3p	0.340	0.375	7.69	8.47	155	20.13

3. Literature

(1) Sanchez, J. G.; Okreglicka, K.; Chandrasekaran, V.; Welker, J. M.; Sundquist, W. I.; Pornillos, O. The tripartite motif coiled-coil is an elongated antiparallel hairpin dimer. *Proc. Natl. Acad. Sci. U. S. A.* **2014**, *111* (7), 2494–2499.

(2) Liu, H.; Naismith, J. H. An efficient one-step site-directed deletion, insertion, single and multiple-site plasmid mutagenesis protocol. *BMC Biotechnol.* **2008**, *8*, 91.

(3) Schmidt, T.; Wälti, M. A.; Baber, J. L.; Hustedt, E. J.; Clore, G. M. Long distance measurements up to 160 Å in the GroEL tetradecamer using Q-band DEER EPR spectroscopy. *Angew. Chem., Int. Ed.* **2016**, *55* (51), 15905–15909.

(4) Schiemann, O.; Heubach, C. A.; Abdullin, D.; Ackermann, K.; Azarkh, M.; Bagryanskaya, E. G.; Drescher, M.; Endeward, B.; Freed, J. H.; Galazzo, L.; Goldfarb, D.; Hett, T.; Esteban Hofer, L.; Fábregas Ibáñez, L.; Hustedt, E. J.; Kucher, S.; Kuprov, I.; Lovett, J. E.; Meyer, A.; Ruthstein, S.; Saxena, S.; Stoll, S.; Timmel, C. R.; Di Valentin, M.; Mchaourab, H. S.; Prisner, T. F.; Bode, B. E.; Bordignon, E.; Bennati, M.; Jeschke, G. Benchmark test and guidelines for DEER/PELDOR experiments on nitroxide-labeled biomolecules. *J. Am. Chem. Soc.* **2021**, *143* (43), 17875–17890.

(5) Berliner, L. J.; Eaton, G. R.; Eaton, S. S. *Distance measurements in biological systems by EPR:Chapter 2 Relaxation times of organic radicals and transition metal ions 29-154. Sandra S. Eaton and Gareth R. Eaton*, Vol. 19; Springer US, 2002. DOI: 10.1007/b111467.
(6) Jeschke, G.; Chechik, V.; Ionita, P.; Godt, A.; Zimmermann, H.; Banham, J.; Timmel, C. R.; Hilger, D.; Jung, H. DeerAnalysis2006—a comprehensive software package for analyzing pulsed ELDOR data. *Appl. Magn. Reson.* **2006**, *30* (3-4), 473–498.
(7) Fábregas Ibáñez, L.; Jeschke, G.; Stoll, S. DeerLab: a comprehensive software package for analyzing dipolar electron paramagnetic resonance spectroscopy data. *Magn. Reson.* **2020**, *1* (2), 209–224.
(8) Fábregas-Ibáñez, L.; Jeschke, G.; Stoll, S. Compactness regularization in the analysis of dipolar EPR spectroscopy data. *J. Magn. Reson.* **2022**, *339*, 107218.
(9) Fábregas-Ibáñez, L.; Tessmer, M. H.; Jeschke, G.; Stoll, S. Dipolar pathways in dipolar EPR spectroscopy. *Phys. Chem. Chem. Phys.* **2022**, *24* (4), 2504–2520.
(10) Tessmer, M. H.; Stoll, S. chiLife: An open-source Python package for in silico spin labeling and integrative protein modeling. *PLoS Comput. Biol.* **2023**, *19* (3), e1010834.
(11) Jeschke, G. MMM: A toolbox for integrative structure modeling. *Protein Sci.* **2018**, *27* (1), 76–85.
(12) Polyhach, Y.; Bordignon, E.; Jeschke, G. Rotamer libraries of spin labelled cysteines for protein studies. *Phys. Chem. Chem. Phys.* **2011**, *13* (6), 2356–2366.
(13) Tessmer, M. H.; Canarie, E. R.; Stoll, S. Comparative evaluation of spin-label modeling methods for protein structural studies. *Biophys. J.* **2022**, *121* (18), 3508–3519.
(14) Kavan, D.; Man, P. MSTools—Web based application for visualization and presentation of HXMS data. *Int. J. Mass Spectrom.* **2011**, *302* (1-3), 53–58.
(15) Jeschke, G.; Polyhach, Y. Distance measurements on spin-labelled biomacromolecules by pulsed electron paramagnetic resonance. *Phys. Chem. Chem. Phys.* **2007**, *9* (16), 1895–1910.
